# MITF – A controls branching morphogenesis and nephron endowment

**DOI:** 10.1371/journal.pgen.1007093

**Published:** 2017-12-14

**Authors:** Aurélie Phelep, Denise Laouari, Kapil Bharti, Martine Burtin, Salvina Tammaccaro, Serge Garbay, Clément Nguyen, Florence Vasseur, Thomas Blanc, Sophie Berissi, Francina Langa-Vives, Evelyne Fischer, Anne Druilhe, Heinz Arnheiter, Gerard Friedlander, Marco Pontoglio, Fabiola Terzi

**Affiliations:** 1 INSERM U1151—CNRS UMR 8253, Université Paris Descartes, Institut Necker Enfants Malades, Département « Croissance et Signalisation », Hôpital Necker Enfants Malades, Paris, France; 2 Unit on Ocular and Stem Cells Translational Research National Eye Institute, National Institutes of Health, Bethesda, MD, United States of America; 3 INSERM U1016—CNRS UMR 8104, Université Paris Descartes, Institut Cochin, Paris, France; 4 Centre d’Ingénierie Génétique Murine, Institut Pasteur, Paris, France; 5 Scientist Emeritus, National Institute of Neurological Disorders and Stroke, National Institutes of Health, 10 Center Drive, Bethesda, MD, United States of America; Universite de Nice, FRANCE

## Abstract

Congenital nephron number varies widely in the human population and individuals with low nephron number are at risk of developing hypertension and chronic kidney disease. The development of the kidney occurs *via* an orchestrated morphogenetic process where metanephric mesenchyme and ureteric bud reciprocally interact to induce nephron formation. The genetic networks that modulate the extent of this process and set the final nephron number are mostly unknown. Here, we identified a specific isoform of MITF (MITF-A), a bHLH-Zip transcription factor, as a novel regulator of the final nephron number. We showed that overexpression of MITF-A leads to a substantial increase of nephron number and bigger kidneys, whereas *Mitfa* deficiency results in reduced nephron number. Furthermore, we demonstrated that MITF-A triggers ureteric bud branching, a phenotype that is associated with increased ureteric bud cell proliferation. Molecular studies associated with an *in silico* analyses revealed that amongst the putative MITF-A targets, *Ret* was significantly modulated by MITF-A. Consistent with the key role of this network in kidney morphogenesis, *Ret* heterozygosis prevented the increase of nephron number in mice overexpressing MITF-A. Collectively, these results uncover a novel transcriptional network that controls branching morphogenesis during kidney development and identifies one of the first modifier genes of nephron endowment.

## Author summary

The number of nephrons, the functional unit of kidney, varies widely among humans. Indeed, it has been shown that kidneys may contain from 0.3 to more than 2 million of nephrons. Nephrons are formed during development *via* a coordinated morphogenetic program in which the metanephric mesenchyme reciprocally and recursively interacts with the ureteric bud. The fine-tuning of this cross-talk determines the final number of nephrons. Strong evidence indicates that suboptimal nephron endowment is associated with an increased risk of hypertension and chronic kidney disease, a major healthcare burden. Indeed, chronic kidney disease is characterized by the progressive decline of renal function towards end stage renal disease, which occurs once a critical number of nephrons has been lost. Elucidating the molecular mechanisms that control nephron endowment is, therefore, a critical issue for public health. However, little is known about the factors that determine the final number of nephrons in the healthy population. Our data showed that nephron endowment is genetically predetermined and identified *Mitfa*, a bHLH transcription factor, as one of the first modifiers of nephron formation during kidney development. By generating an allelic series of transgenic mice expressing different levels of MITF-A, we discovered that MITF-A promotes final nephron endowment. In addition, we elucidated the molecular mechanisms by which MITF-A promotes nephron formation and identified RET as one of the critical effectors.

## Introduction

For decades, it was believed that the number of nephrons in the kidneys does not vary among normal individuals. However, several studies performed over the last 30 years have clearly demonstrated that the number of nephrons varies widely among human populations and even among healthy individuals of the same ethnicity [1]. In fact, kidneys may contain from 0.3 to more than 2 million nephrons. Strong evidence indicates that suboptimal nephron endowment is associated with an increased risk for developing essential hypertension and chronic kidney disease (CKD) [1,2]. CKD is characterized by a progressive decline in renal function eventually leading to end stage renal disease which occurs when a critical number of nephrons has been lost, irrespective of the cause of the renal damage. Over 10% of the adult population is estimated to suffer from CKD [3]. Elucidating the cellular events and genetic networks that control nephron endowment is, therefore, a critical issue for understanding the predisposition to CKD and consequently for public health.

Little is known about the factors that determine nephron number in the normal population. Individual nephrons are formed during fetal development *via* an orchestrated morphogenetic process that is characterized by the reciprocal interaction between the metanephric mesenchyme (MM) and the ureteric bud (UB) [4,5] and that critically determines the final nephron number [6]. In fact, subtle modifications in the efficiency and/or accuracy of this process can lead to renal hypoplasia [7]. Epidemiological studies have indicated that both environmental and genetic factors play critical roles in nephron development [6,8,9]. Global caloric and protein restriction as well as iron or vitamin A deficiency result in reduced nephron number [9]. Similarly, reduced birth weight either due to premature birth or intrauterine growth retardation correlates with low nephron number [10]. On the other hand, it has been shown that hypomorphic variants of *PAX2* and *RET*, two genes encoding critical players of branching morphogenesis, are associated with a 10% decrease in renal volume at birth [11,12]. Conversely, it has been reported that a relatively common variant of *ALDH1A2*, a gene encoding an enzyme involved in retinoic acid metabolism, is associated with a 22% increase in kidney size at birth [13].

Microphthalmia-associated transcription factor (MITF) is a member of the basic/helix-loop-helix/leucine zipper (b-HLH-Zip) family of transcription factors [14]. To date, at least nine isoforms of MITF have been identified that arise from the use of alternative promoters transcribing different (protein coding) first exons, leading to the expression of proteins carrying distinct N terminal portions [15,16]. Some isoforms, including MITF-A, are widely expressed, while others are cell-type-specific. MITF is known to be essential for the development and function of different cell lineages such as pigmented cells, mast cells and osteoclasts [15,17]. In addition, MITF plays a role in pathophysiological events, such as in cardiac growth and hypertrophy [18], B cell homeostasis [19], and melanoma proliferation and invasiveness [20]. Recently, MITF has also been shown to be crucial for renal pathology since *MITF* variants were shown to affect the progression of renal disease [21] or influence the occurrence of renal carcinoma [22]. In this context, our group has discovered that a hypomorphic variant in the 5' UTR of MITF-A predisposes FVB/N mice to develop renal lesions after experimental nephron reduction [21].

Here, in order to improve our knowledge on MITF-A function in renal pathophysiology, we generated several lines of transgenic mice expressing different levels of MITF-A. We discovered that MITF-A regulates the final nephron number by modulating UB branching. Enhanced UB branching occurs *via* an increase of cell proliferation involving key developmental targets, including RET. Collectively, our results reveal a novel actor of kidney development and uncover a novel function of MITF-A.

## Materials and methods

### Generation of kidney-specific MITF-A overexpressing mice

The Ksp-cadherin, FLAG-MITF-A fusion gene was generated by cloning the full-length mouse MITF-A tagged at the 5′end by a FLAG epitope into the Ksp/BGH/link plasmid (kindly provided by Peter Igarashi) containing the minimal Ksp-cadherin promoter followed by the beta-globin enhancer element [23]. The poly(A) sequence for the human beta-globin gene was isolated and cloned into the Ksp-cadherin-FLAG-MITF-A plasmid. Transgenic mouse lines were generated by microinjecting the purified DNA construct into fertilized mouse oocytes derived from a pure genetic background FVB/N. Twenty FLAG-MITF-A transgenic founder lines were identified by PCR analysis of tail DNA using primers specific to the MITF-A transgene (**S3 Table**).

### Generation of *Mitfa*^*-/-*^ mutant mice

To generate the targeting construct, the potential *Mitfa* promoter/*Mitfa* exon and its flanking regions (15,942 bp) were cloned using plasmid rescue from BAC RP23-77E9. A floxed neomycin resistance expression cassette flanked by 200 bp of sequence flanking the *Mitfa* promoter/*Mitfa* exon was used to replace 5,956 bp of the putative *Mitfa* promoter/*Mitfa* exon from the above plasmid and used for standard targeting of LC3 ES cells (genotype [C57BL/6Nx129S6]F1). A correctly targeted ES cell colony was used to generate chimeric animals. Of several germline-transmitting lines, one was selected and crossed with C57BL/6JN/129S4-Prm1-Cre deleter mice (Jackson Laboratories, stock 003328, backcrossed twice to C57BL/6J) to remove the floxed neomycin cassette. Offspring lacking the neo-cassette were backcrossed to C57BL/6J mice for nine generations and then bred to homozygosity. Wild-type littermates were always used as controls. The primers used for genotyping are indicated in **S3 Table**.

### Generation of MITF-A^wt/tgMITF-A^*; Ret*^*wt/-*^ *double transgenic* mice

To generate MITF-A^wt/tgMITF-A^*;Ret*^*wt/-*^ mice, heterozygous FVB/N mice overexpressing MITF-A (see above) were crossed with heterozygous C57BL/6 *Ret* knockout mice [38]. Double heterozygous offsprings seemed normal and were born in the expected proportion.

### Experimental protocols

Animals were fed ad libitum and housed at constant ambient temperature in a 12-hour light cycle. Animal procedures were approved by the Departmental Director of “Services Vétérinaires de la Préfecture de Police de Paris” and by the ethical committee of the Paris Descartes University as well as by the NIH/NINDS intramural program.

For the post-natal characterization of the MITF-A transgenic line, transgenic mice and wild-type littermates were sacrificed 2, 4, 6 and 12 months after birth (n = 5 to 12 for each genotype and time point). For renal function studies, urine samples were collected one week before sacrifice using metabolic cages over the course of 24 hours and blood samples were obtained at time of sacrifice (n = 4 to 6 for each genotype and time point) and kidneys dissected for appropriate studies. In addition, liver, spleen, heart, lung and brain were removed at 2 months for mRNA studies. For developmental studies, except where stated otherwise, the number of kidneys removed from E13.5 to P0 ranged between 14 and 25 for each genotype. For genetic interaction between *Mitfa* and *Ret* studies, mice were sacrificed at 3 weeks, kidneys were removed and the number of glomeruli was counted in females, exclusively (n = 3 to 7 for each genotype, i.e. MITF-A^*wt/wt*^*;Ret*^*wt/wt*^, MITF-A^wt/tgMITF-A^*;Ret*^*wt/wt*^, MITF-A^*wt/wt*^*;Ret*^*wt/-*^, *and* MITF-A^wt/tgMITF-A^;*Ret*^*wt/-*^*)*.

### Urine and plasma analyses

Plasma urea levels and urinary albumin, protein and creatinine levels were measured using an Olympus multiparametric autoanalyser (Instrumentation Laboratory).

### Renal morphology

For morphological analysis, kidneys were fixed in 4% paraformaldehyde, paraffin embedded, and 4-μm sections were stained with periodic acid Schiff (PAS) or Hematoxylin & Eosin (HE). A pathologist, blinded to the nature of the group, examined and evaluated all the sections. For morphometric analysis, glomerular and tubular surfaces were measured on PAS-stained sections, using a Nikon digital camera Dx/m/1200 and Lucia software (Laboratory Imaging Ltd., Prague, Czech Republic). Twenty randomly selected microscopic fields of the cortex were studied (X200). At least 20 glomeruli were analyzed for each animal. Tubular morphometric analysis was performed on sagittal kidney sections. The area of external profile of proximal tubules and the area of the lumen were measured, and the epithelial surface was calculated as the difference between these two areas. At least 35 tubular cross sections were analyzed for each animal. Tubular cell size was evaluated on the same tubular cross sections and was calculated as the ratio of epithelial surface to nuclei number for each tubular section.

### Determination of glomeruli number

Kidneys of two month-old mice were de-capsulated and macerated in 5N HCL for 30 minutes at 37°C. After rinsing with distilled water, the suspension was stored overnight at 4°C. The flask was shaken on the following day and the tubules and glomeruli were suspended in 25 ml of distilled water. Glomeruli were counted on five different 250 μl aliquots using a Nikon Eclipse E800 microscope.

### Whole mount UB immunofluorescence

Kidneys from E13.5 mice were dissected and fixed in cold methanol for 15 minutes. After washing in PBS for 15 minutes, the entire kidneys were treated with 0.1% Triton in PBS for 2 hours. To visualize the branches and tips of the ureteric tree, kidneys were incubated overnight with primary mouse antibody against Calbindin-D28K (Sigma-Aldrich, 1:200), a calcium-binding protein expressed in the ureteric epithelium. Metanephroi were then washed in PBS and incubated with the secondary antibody AlexaFluor 555 goat anti-mouse IgG (Invitrogen, Molecular Probes, 1:100). The number of UB tips was counted using a Nikon Eclipse E800 microscope.

### Cell proliferation and apoptosis assays

For cell proliferation, 4-μm sections of paraffin-embedded kidneys were incubated with a rabbit anti-phospho-histone H3 antibody (Millipore) at 1:200 and a mouse anti-PCNA antibody (DAKO) at 1:50, followed by the appropriate secondary antibody. The number of proliferating cells was determined in UB and expressed as the number of pH3- or PCNA- positive cells per UB structure on whole kidney sections.

Apoptosis was detected in 4-μm sections of paraffin-embedded kidneys by TUNEL assay using the In Situ Cell Death Detection kit (Roche) according to the manufacturer’s protocol. The number of apoptotic cells was determined as the number of TUNEL–positive nuclei per microscopic field in whole kidney sections. Ten microscopic fields were scored for each kidney in 5 embryos of each genotype.

### MITF-A immunohistochemistry

Four μm sections were retrieved with Tris-EDTA (TE) buffer (pH = 9.0) at 90°C for 20 minutes, then incubated first overnight at 4°C with a rabbit anti-MITF-A antibody [21] 1/200, then with a secondary biotinylated anti-rabbit antibody (Vector) 1/500, followed by streptavidine-peroxidase (Dako) 1/500. Staining was revealed by 3–3’-diaminobenzidine (DAB).

### *In situ* hybridization

*In situ* hybridization was carried out on 10-μm cryosections from OCT embedded kidneys frozen in isopentane solution under liquid nitrogen. Digoxigenin-dUTP–labeled sense and antisense RNA probes were synthesized using Roche reagents. The following cDNA templates were used: *Ret*, *Wnt11*, *Pax2*, *BMP7*, *Wnt9b* and *Spry1* (a kind gift from Isabelle Gross, INSERM U682). An RNA probe against MITF-A was obtained from cDNA encompassing both exon 1A and exon 1B. The resulting RNA probe could hybridize with the endogenous MITF-A and the MITF-A transgene and potentially with other MITF isoforms containing exon 1B (MITF-H, MITF-C, MITF-J, MITF-B, MITF-Mc). We designed this probe, since we failed to get a specific staining with a shorter probe (177 nucleotides) that overlaps only with MITF-A sequence. Cryosections were fixed with 4% paraformaldehyde for 10 min, acetylated for 10 min and then pre-hybridized for 3 hours. Hybridization was performed overnight at 60°C in the presence of 1 μg/ml for each RNA probe. After successive washings at 60°C in 2X and 0.2X SCC buffer, the sections were washed in Tris buffer pH 7.6 (0.1 M Tris, 0.15 M NaCl) incubated in 2% blocking reagent solution (Roche) for 3 hours, and then incubated overnight with anti-digoxigenin antibody (alkaline phosphatase-conjugated Fab, Roche, 1:1,500). Sections were successively washed in Tris buffer, pH 7.6 and in Tris buffer, pH 9.5 (0.1 M Tris, 0.1M NaCl, 50 mM Mgcl2) and finally incubated with NBT/BCIP AP substrate solution (Roche).

For colocalization staining, the sections were successively fixed in 4% paraformaldehyde and incubated overnight with a rabbit antibody anti-laminin (Sigma-Aldrich), at 1:200, followed by an Alexa-Fluor 555 donkey anti-rabbit antibody (Invitrogen, Molecular Probes), at 1/200.

### Western blot analysis

MITF-A immunoblotting was performed on nuclear extracts obtained after a 100,000 g ultracentrifugation of kidneys homogenized in 10 mM Hepes /1.7 M sucrose buffer (pH 7.9) supplemented with DTT (0.5 mM), spermine (0.15 mM), spermidine (0.5 mM), benzamidine (2 mM), anti-protease and anti-phosphatase inhibitors. The nuclear pellet was suspended in 8 M urea buffer and centrifuged. The supernatant was assayed for MITF-A immunoblotting using a rabbit antibody raised against a recombinant GST-MITF-A protein [21]. A polyclonal antibody against Lamin-A/C (Epitomics) was used as a control for nuclear proteins.

### Quantitative RT-PCR

Kidneys were incubated in RNA later (Ambion) overnight and stored at -80°C until extraction. Total RNA was extracted from embryonic kidneys using RNeasy Micro Kit from Qiagen. RNAs were DNase treated (DNase I RNase-free, Qiagen) and reverse transcribed according to the manufacturer's protocols (Superscript II, Invitrogen). Quantitative RT-PCR was carried out using an ABI PRISM 7700 Sequence Detection system (Applied Biosystems). HPRT was used as the normalization control. The primers used (Eurogentec) are described in **S3 Table**.

### *In silico* analysis

Positional weight matric (PWM) was generated from 47 published functional MITF binding sites [27] and by using the program MEME 3.5.0 (http://meme.nbcr.net/meme/) to search both strands for putative motifs. The informative portion of the aligned sequences was converted into a PWM by assigning to each position/nucleotide the frequency of that given nucleotide in that given position. PWM sequence logo representation was created by submitting MEME informative sequences to WebLogo (http://weblogo.berkeley.edu/) The identification of the conserved predicted MITF-A binding sites among the 102 kidney developmental genes (MGI abnormal kidney development data base) was then carried out as described previously [26].

### Data analysis and statistics

Data are expressed as means ± SEM. Differences between the experimental groups were evaluated using ANOVA, and, when significant (*P* < 0.05), followed by the Tukey-Kramer test. When only two groups were compared, the Mann-Whitney test was used. The Pearson’s correlation coefficient was used to test correlation between variables. The statistical analysis was performed using Graph Prism Software.

## Results

### Generation of lines of transgenic mice overexpressing MITF-A

**Fig 1:**
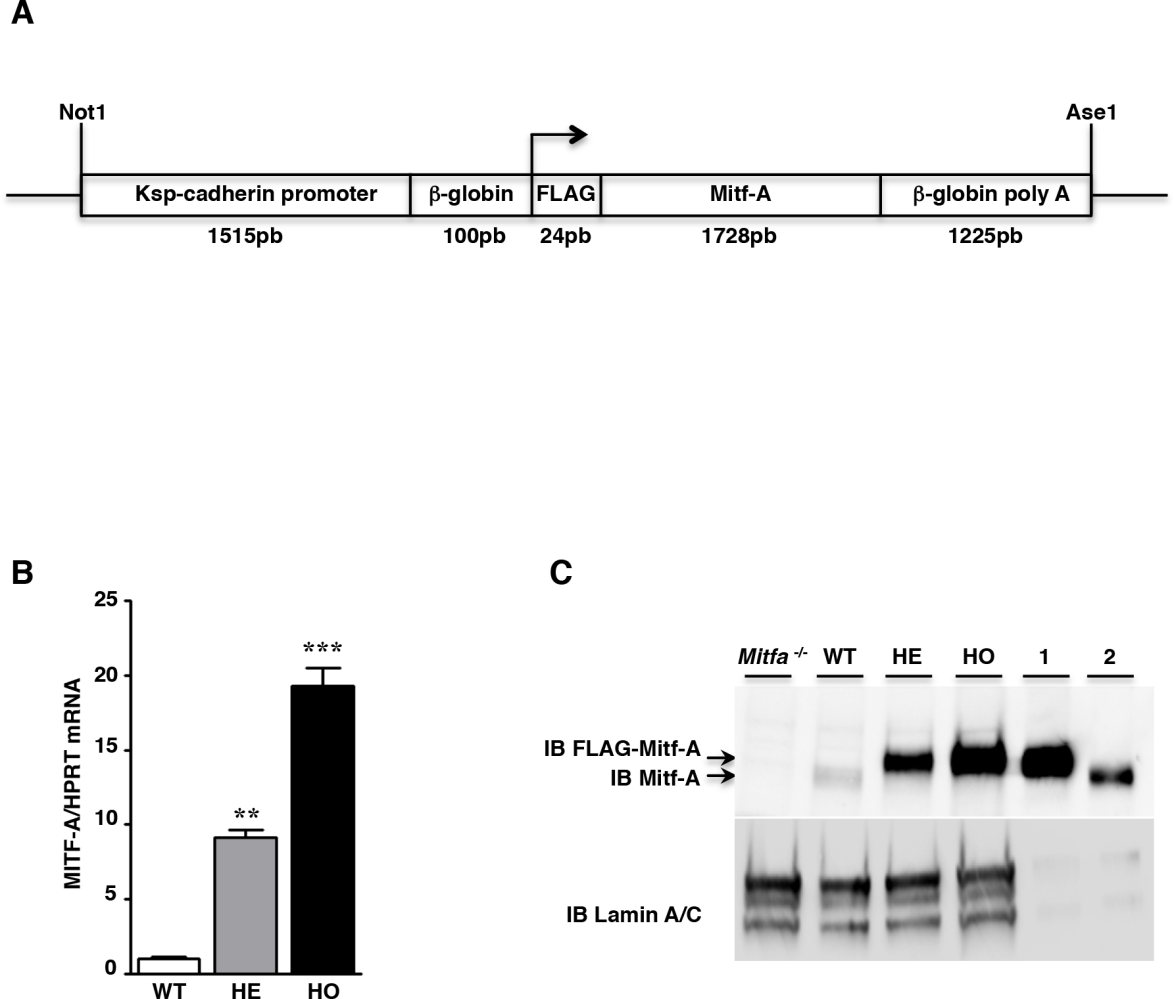
Generation of MITF-A transgenic mice. **A)** Schematic representation of the Ksp-cadherin-FLAG-MITF-A transgene. **B)** *Mitf-A* mRNA expression evaluated by quantitative RT-PCR in kidneys from wild-type (WT), heterozygous (HE) and homozygous (HO) MITF-A transgenic mice (line 42) 2 months after birth. Data are means ± SEM; n = 4–6 per each genotype. ANOVA followed by Tukey-Kramer test; transgenic *versus* wild-type mice: ** P < 0.01, *** P < 0.001. **C)** MITF-A protein expression evaluated by western blot on kidney nuclear protein extracts from WT, HE and HO MITF-A transgenic mice 2 months after birth. This is a representative image of three experiments. Nuclear protein extracts from *Mitfa*^-/-^ kidneys were used as a negative control; crude extracts from renal cells transfected with either FLAG-MITF-A plasmid (lane 1) or MITF-A plasmid (lane 2) were used as a positive control. Lamin A/C was used as control of nuclear protein amount. IB = immunoblot.

In order to elucidate the role of MITF-A in renal pathophysiology, we generated a set of FVB/N transgenic mouse lines (here indicated as MITF-A mice) overexpressing MITF-A in the kidney under the control of the Ksp-cadherin promoter (**Fig 1A**). Twenty founders were identified, and lines were established from each of them. Among them, only three (line 14, 42 and 47) overexpressed MITF-A selectively in the kidney, and not in liver, spleen, heart, lung, or brain (**S1 Fig**). Quantitative RT-PCR showed that *Mitf-A* expression levels varied in the kidneys of the three transgenic lines. The highest level was detected in the kidneys of line 42 heterozygotes and homozygotes, in which *Mitf-A* mRNA was increased 10- and 20-fold, respectively, as compared to kidneys of wild-type littermates (**Fig 1B**). In contrast, the increase was only 5-fold and 3-fold in heterozygotes of line 14 and 47, respectively (**S2A** and **S2D Fig**). Western-blot analysis showed that the levels of the nuclear protein paralleled that of the corresponding mRNA (**Fig 1C**).

### MITF-A overexpression leads to larger kidney size and increased nephron number

**Fig 2:**
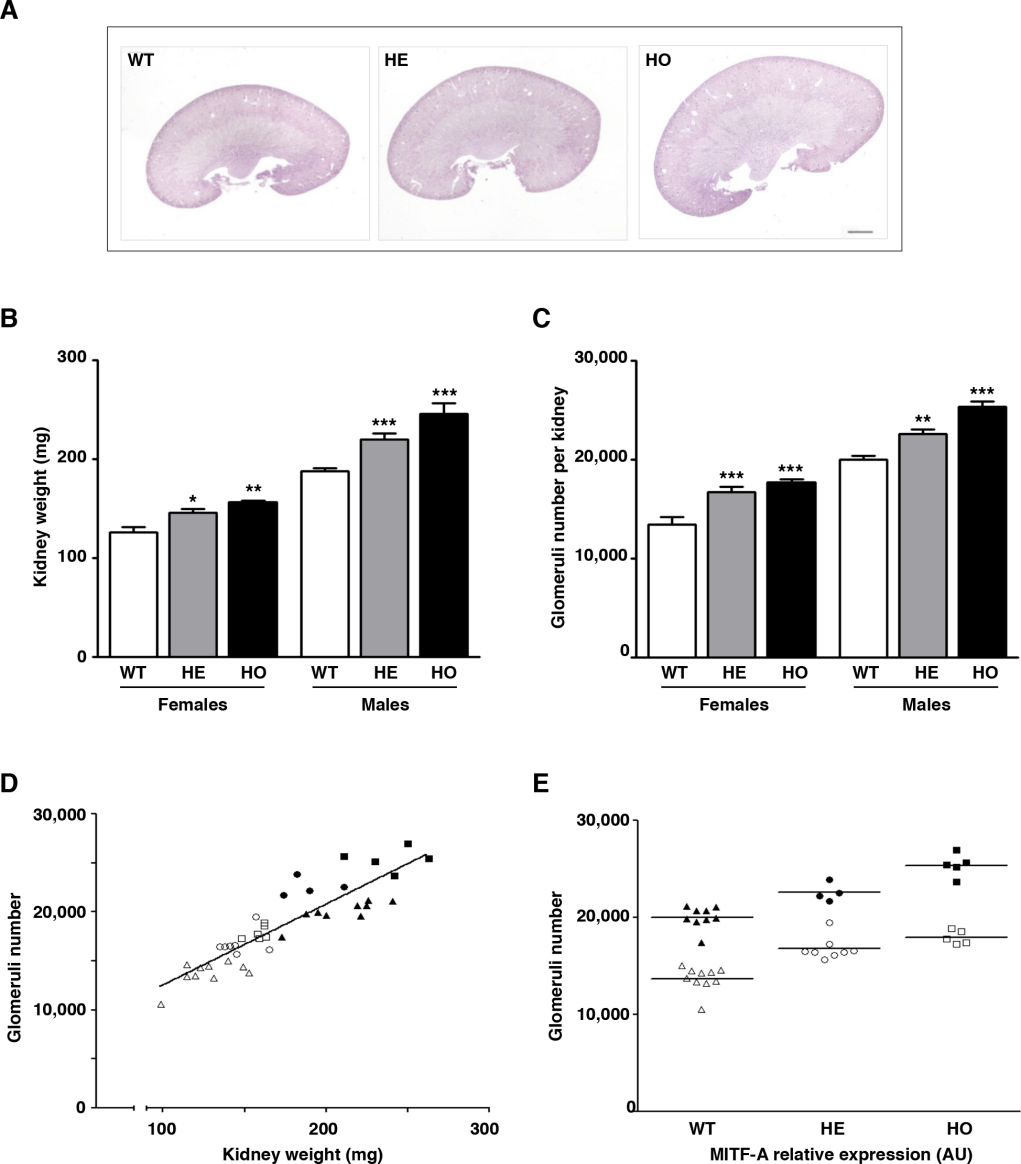
Overexpression of MITF-A results in increased kidney size and glomerular number. **A)** Morphology of kidneys from wild-type (WT), heterozygous (HE) and homozygous (HO) MITF-A transgenic mice of line 42, 2 months after birth. Shown are representative images of at least 10 mice for each genotype. Bar = 100 μm. **B-C)** Kidney weight (KW) (**B)** and glomeruli number per kidney **(C)** in WT, HE and HO MITF-A transgenic females and males. Data are means ± SEM, n = 4–8 per each genotype. ANOVA followed by Tukey-Kramer test; transgenic versus wild-type mice: * P < 0.05, ** P < 0.001, *** P < 0.001. **D)** Correlation between kidney weight and glomeruli number in WT (white and black triangles, in females and males, respectively), HE (white and black circle, in females and males, respectively) and HO (white and black square, in females and males, respectively) MITF-A transgenic mice *(r* = 0.96, P < 0.0001). **E)** Glomeruli number increased proportionally to *Mitf-A* expression levels in both females (white symbols) and males (black symbols).

Transgenic mice overexpressing MITF-A were viable, fertile, and appeared phenotypically normal. Their kidneys, however, were significantly larger than those of wild-type littermates when analyzed two months after birth (**Fig 2A and 2B**). This difference was already detectable at birth, was maintained at least until 12 months of age (**S3 Fig**), and was kidney specific as no differences were detected in body, heart, liver and spleen weights (**Table 1**). Renal histology revealed no gross abnormalities of the cortex/medulla architecture as well as of the glomerular and tubular morphology in MITF-A transgenic mice from 2 to 12 months after birth (**S4A Fig**). Furthermore, no differences in blood urea, albuminuria and urinary protein excretion were found at 2, 4, 6 and 12 months after birth (**S4B Fig**), suggesting that renal function was not affected.

**Table 1:** Characterization of MITF-A transgenic mice.

	BW (g)	KW/BW (mg/g)	HW/BW (mg/g)	LW/BW (mg/g)	SW/BW (mg/g)
*Females*					
WT	19.7 ± 1.0	5.7 ± 0.2	5.1 ± 0.2	51.7 ± 2.8	4.9 ± 0.3
HE	21.5 ± 0.4	6.7 ± 0.3^a^	5.1 ± 0.1	53.8 ± 1.0	5.1 ± 0.2
HO	21.4 ± 0.4	7.3 ± 0.2^c^	5.2 ± 0.1	50.1 ± 1.1	5.1 ± 0.1
*Males*					
WT	27.4 ± 0.5	6.9 ± 0.1	5.1 ± 0.1	50.4 ± 1.1	4.3 ± 0.1
HE	27.3 ± 0.8	8.1 ± 0.2^a^	4.6 ± 0.3	50.9 ± 1.5	4.1 ± 0.2
HO	26.2 ± 0.5	9.4 ± 0.3^c^	5.4 ± 0.5	51.5 ± 0.5	4.5 ± 0.2

Body (BW), kidney (KW), heart (HW), liver (LW) and spleen (SW) weight 2 months after birth. WT: wild-type mice; HE: heterozygous MITF-A transgenic mice; HO: homozygous MITF-A transgenic mice. Data are means ± SEM; n = 4–6 per each genotype. ANOVA followed by Tukey-Kramer test; transgenic *versus* wild-type mice:

^a^ P < 0.05

^c^ P < 0.001.

Kidney overgrowth may result from an increase of either nephron size (hypertrophy) or nephron number. To determine which of these events might account for the increased kidney size in MITF-A transgenic mice, we first performed a morphometric analysis and measured the average surface of tubular and glomerular sections. Our results showed that the average tubular epithelial cell surface (external surface minus lumen surface for individual tubules) was comparable between MITF-A transgenic mice and wild-type littermates two months after birth (**S1 Table**), and the average glomerular surface area was even significantly decreased in MITF-A transgenic mice as compared to wild-type littermates (**S1 Table**). These data argue against a role of hypertrophy in MITF-A induced renal overgrowth. Therefore, we next evaluated the average number of nephrons contained in each kidney. Remarkably, in both males and females, the number of glomeruli was significantly higher (30%) in MITF-A homozygous transgenic kidneys as compared to their wild-type counterparts (**Fig 2C**). A highly significant correlation (*r* = 0.96; P < 0.0001) was found between kidney weight and glomeruli number (**Fig 2D**). Moreover, the number of glomeruli was positively correlated with the different *Mitf-A* expression levels found in wild type, heterozygous and homozygous mice of line 42 (**Fig 2E**), suggesting a role for MITF-A in final nephron endowment. Nevertheless, we could not formally rule out that the biological effect observed in these mice was due to adverse effects of transgene integration in a crucial genomic site. In order to rule out this possibility, we studied the two additional MITF-A overexpressing transgenic mouse lines. Consistent with the fact that these lines expressed MITF-A at lower levels than line 42, the increase in nephron number in heterozygotes was less pronounced (**S2B** and **S2E Fig**). Of note, as in line 42, renal morphology appeared normal in two month-old heterozygous mice from both line 14 and 47 (**S2C** and **S2F Fig**). This finding further supported the notion that transgenic MITF-A levels were correlated with nephron number and excluded the possibility that a specific integration of the transgene accounted for kidney overgrowth.

### *Mitfa* inactivation results in reduced nephron number

**Fig 3:**
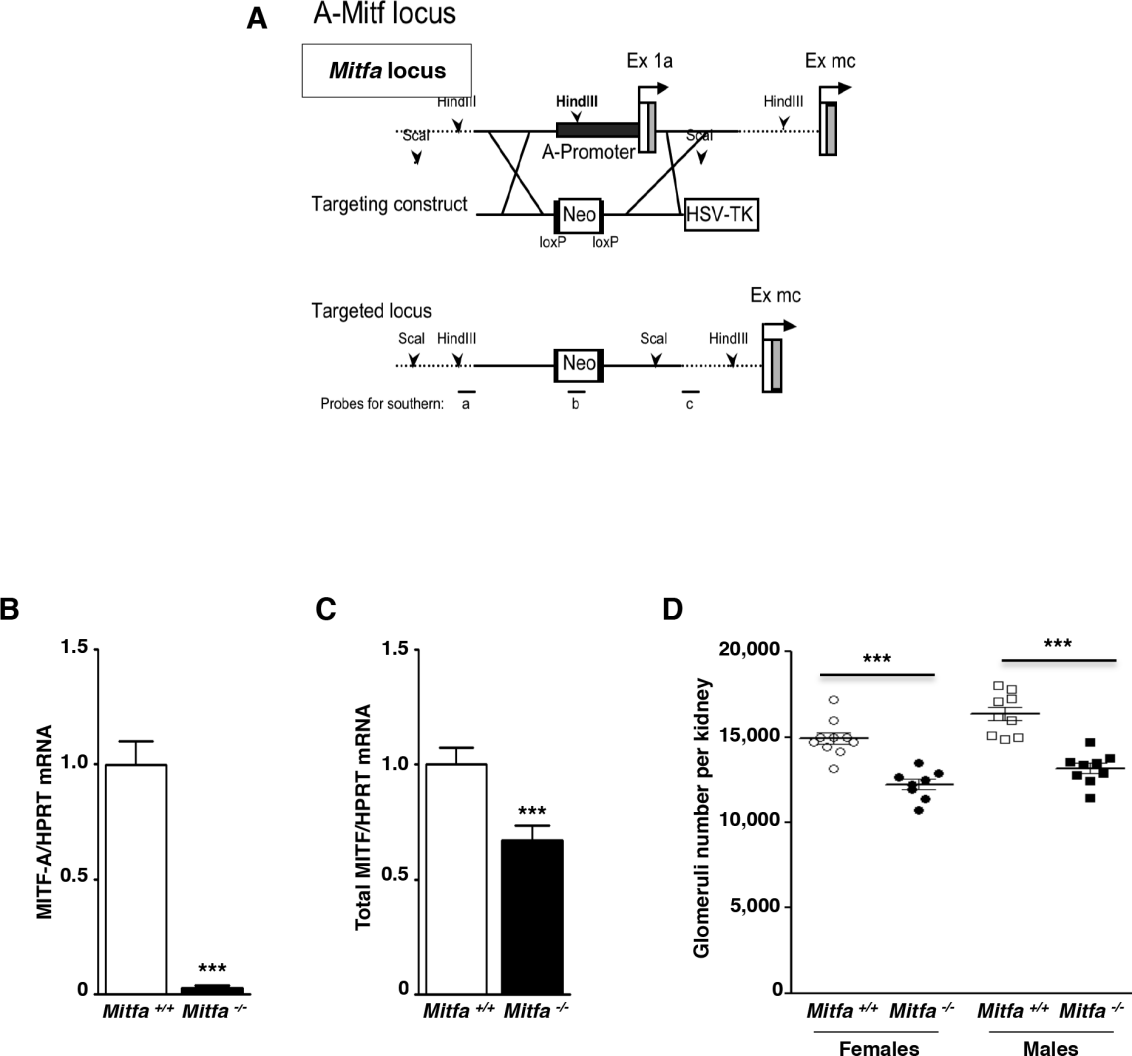
*Mitfa* inactivation results in reduced glomeruli number. **A)** Schematic representation of the targeting strategy used to inactivate *Mitfa*. **B-C)** *Mitf-A* **(B)** and total *Mitf* mRNA **(C)** expression evaluated by quantitative RT-PCR in kidneys from 2 months-old *Mitfa*^*+/+*^ and *Mitfa*^*-/-*^ mice. **D)** Glomerular number in kidneys from 2 months-old *Mitfa*^*+/+*^ and *Mitfa*^*-/-*^ mice. Data are means ± SEM, n = 8–10 per each genotype. Mann-Whitney test; *Mitfa*^*-/-*^ *versus Mitfa*^*+/+*^: *** P < 0.001.

To further assess the role of MITF-A in nephron endowment, we produced a mouse line carrying a specific deletion of the *Mitfa* promoter by homologous recombination (**Fig 3A**). As expected, *Mitf-A* mRNA expression was negligible in kidneys of *Mitfa*^*-/-*^ mice (**Fig 3B**) and total *Mitf* mRNA expression levels (that include all the isoforms) were significantly decreased (**Fig 3C**), indicating that the loss of the MITF-A isoform was not compensated by an increase of any of the other isoforms. Homozygous null *Mitfa* mice were fertile, had a normal phenotype and were indistinguishable from their wild-type littermates. Consistent with the above results obtained with MITF-A overexpressing transgenic mice, however, the number of their glomeruli was significantly reduced (20%) as compared to wild-type littermates (**Fig 3D**).

### MITF-A affects branching morphogenesis in transgenic mice

**Fig 4:**
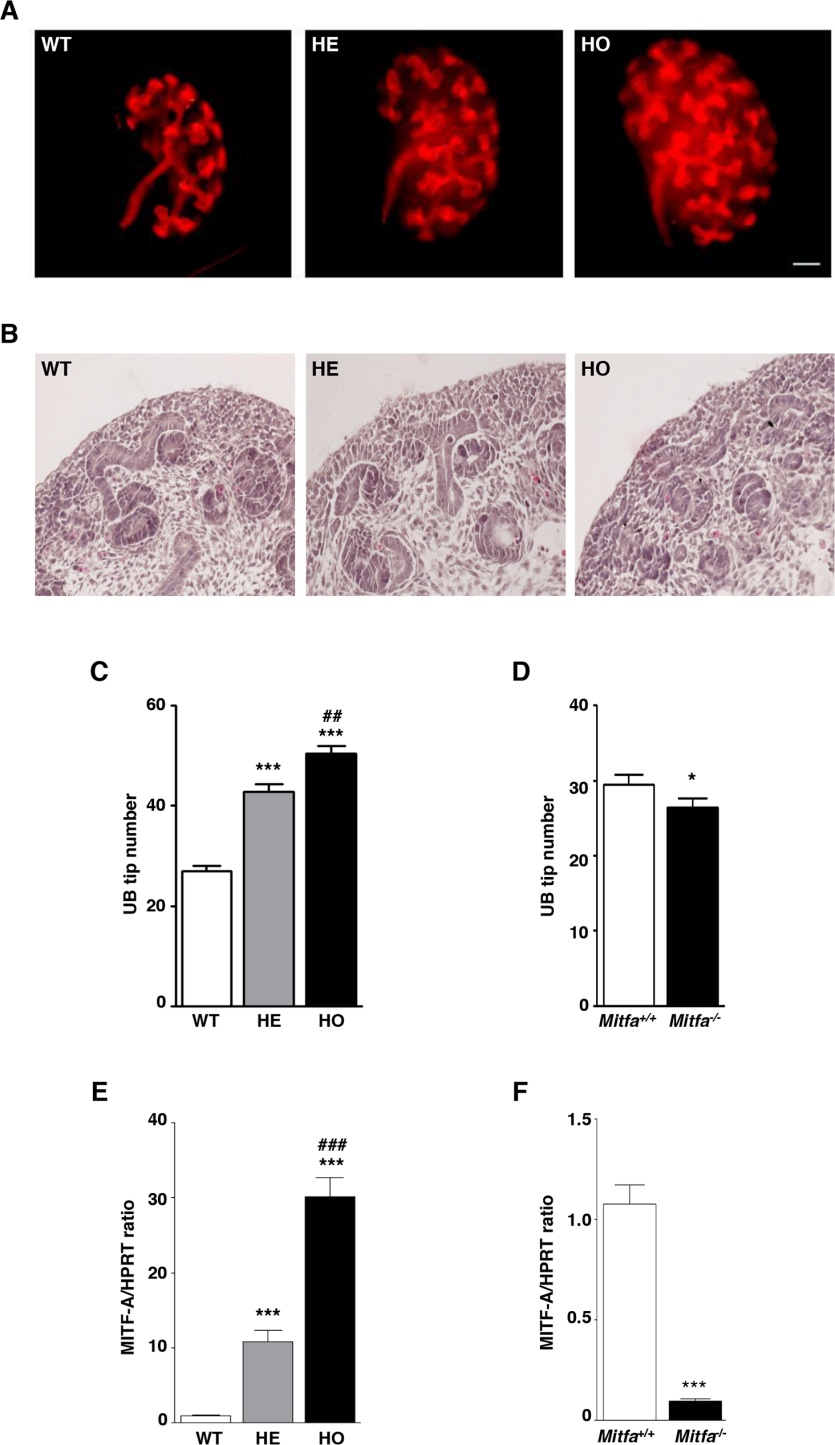
MITF-A modulates kidney branching morphogenesis. **A)** Whole mount E13.5 metanephroi in wild-type (WT), heterozygous (HE) and homozygous (HO) MITF-A transgenic embryos (line 42) after staining with anti-Calbindin antibody. These are representative images of at least 6 embryos for each genotype. Bar = 100 μm. **B)** Morphology of kidneys in WT, HE and HO MITF-A transgenic embryos at E13.5. These are representative images of at least 6 embryos for each genotype. **C-D)** Ureteric bud (UB) branching, as assayed by counting the number of UB tips in (**C**) WT (n = 17), HE (n = 14) and HO (n = 25) MITF-A transgenic embryos and (**D**) *Mitfa*^*+/+*^ (n = 15) and *Mitfa*^*-/-*^ (n = 20) embryos at E13.5. **E-F)** *Mitf-A* mRNA expression evaluated by quantitative RT-PCR in kidneys from (**E**) WT, HE and HO MITF-A transgenic embryos (n = 7–8 per each genotype) and (**F**) *Mitfa*^*+/+*^ and *Mitfa*^*-/-*^ embryos (n = 7 and 3 per genotype, respectively) at E 13.5. Data are means ± SEM. For transgenic MITF-A mice: ANOVA followed by Tukey-Kramer test; transgenic *versus* wild-type mice: *** P < 0.001, HE v*ersus* HO MITF-A transgenic mice: ## P < 0.01, ### P < 0.01. For *Mitfa* knockout mice: Mann-Whitney test; *Mitfa*^*-/-*^ versus: *Mitfa*^*+/+*^: * P < 0.05, *** P < 0.001.

The final number of nephron is determined by the fine-tuning of mesenchyme/ureteric bud (UB) crosstalk. Since the Ksp-cadherin promoter drives the expression of the transgene in the UB [23], we hypothesized that MITF-A may affect branching morphogenesis. To assess this, we monitored branching morphogenesis in mice of line 42 during embryogenesis. Using calbindin-1 staining, we first showed that the overall branching pattern appeared normal. However, the number of branchings was significantly increased in transgenic embryos (**Fig 4A**), while the global architecture of developing kidneys was similar between MITF-A transgenic and wild-type embryos from E13.5 to P14 (**Fig 4B**). Notably, at E13.5, metanephroi of MITF-A transgenic embryos exhibited the typical un-induced metanephric mesenchyme and growing branch tips in the external portion of the cortex (**Fig 4B**). Quantitative analysis confirmed that the number of UB tips was significantly higher in MITF-A transgenic kidneys of embryos from line 42 as compared to wild-type controls **(Fig 4C** and **S5A Fig**). These results were confirmed in transgenic line 47, in which the number of UB tips was 36.3 ± 2.1 in heterozygous transgenics and 26.3 ± 1.7 in wild-type littermates, respectively (P < 0.001). On the other hand, in *Mitfa*^*-/-*^ embryos, the number of UB tips was mildly but significantly reduced at E13.5 as compared to that in wild-type littermates (**Fig 4D** and **S5B Fig**). Quantitative RT-PCR confirmed the increase of MITF-A mRNA in metanephroi of E13.5 MITF-A overexpressing embryos as compared to wild-type controls (**Fig 4E**). On the contrary, the expression of *Mitf-A* was negligible in *Mitfa*^*-/*-^ embryonic kidneys (**Fig 4F**). Taken together, these data point to an unexpected role for MITF-A in branching morphogenesis.

### MITF expression during nephrogenesis

**Fig 5:**
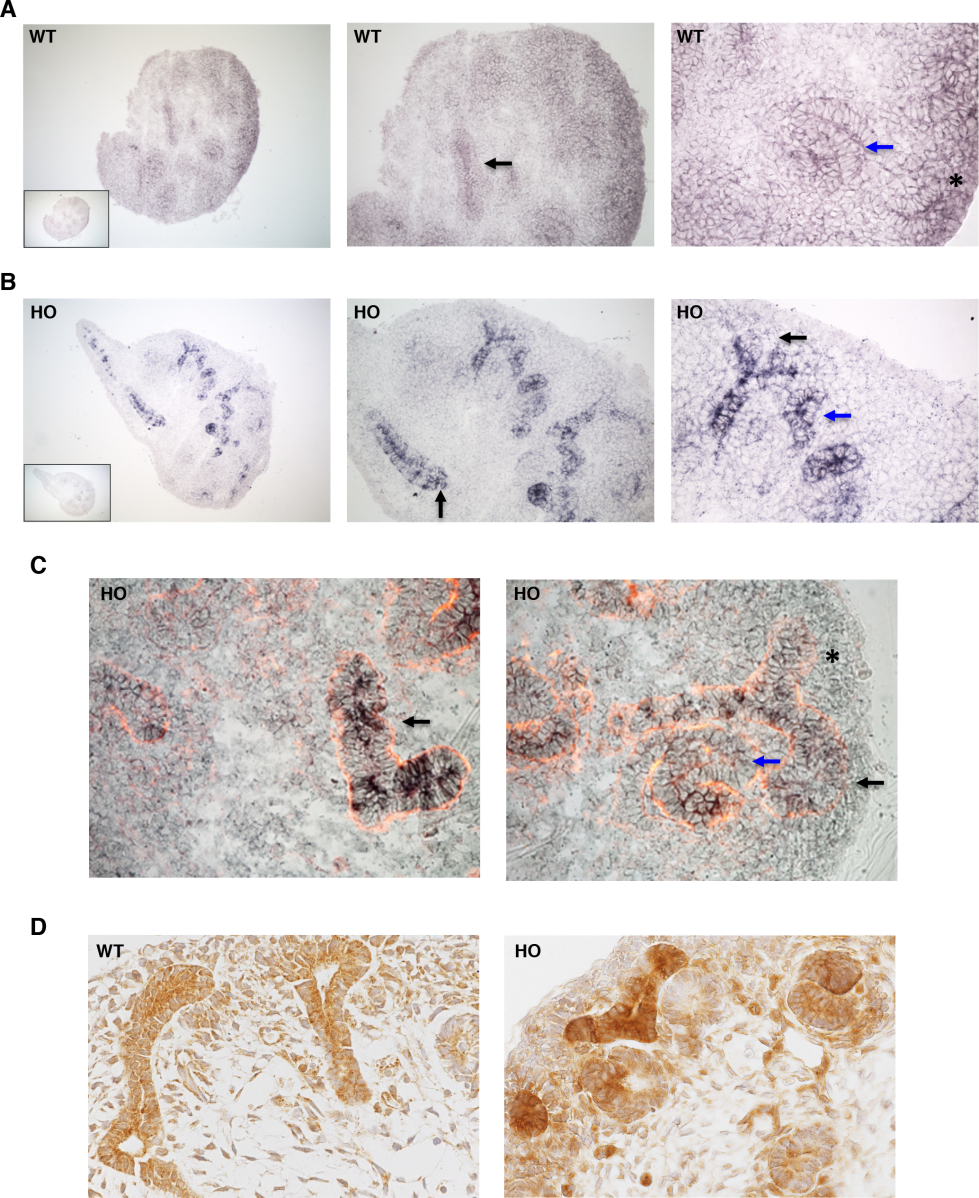
Expression pattern of MITF-A during kidney development. **A-B)** *In situ* hybridization of *Mitf-A* of E13.5 kidneys from wild-type (WT) and homozygous (HO) MITF-A transgenic embryos using an antisense RNA probe directed against a sequence encompassing exon 1A, specific for *Mitf-A*, and exon 1B common to *Mitf-A*, *Mitf-H*, *Mitf-C*, *Mitf-J* and *Mitf-Mc* isoforms. The inset shows the staining of E13.5 kidneys using the sense RNA probe. Magnifications are X100 (left panels), X200 (middle panels) and X400 (right panels). In WT kidneys **(A)** a weak staining is observed in branches of UB (black arrow), in S-shaped body (blue arrow) and in metanephric mesenchyme (asterisk). Consistent with the use of the Ksp-cadherin promoter, the signal in MITF transgenic kidneys **(B)** was strongly increased in UB and tips (black arrow), in ureteric tip (black arrow) and to a lesser extent in S-shaped body (blue arrow). **C)** *In situ* hybridization of *Mitf-A* in transgenic HO kidneys after laminin immunohistochemistry (red). Note *Mitf* expression in ureteric bud and tip (black arrow), in and S-shaped body (blue arrow). Magnification X400. Sections are representative images of 4 kidneys per genotype. **D**) Immunostaining of MITF-A in WT and HO MITF-A transgenic metanephroi at E13.5. Note the increase of MITF-A expression in UB stalks, tips and S-bodies. Magnification X400.

Since our data revealed MITF-A as a novel actor in nephrogenesis, we next tried to define its expression pattern and its effect on other MITF isoforms. *In situ* hybridization revealed that in wild type embryos at E13.5, *Mitf-A* is expressed in the UB, the mesenchyme and in S-shaped bodies (**Fig 5A**). In transgenic metanephroi, *Mitf-A* staining was markedly increased, particularly in UB branches (**Fig 5B**) as expected from the pattern of expression driven by the Ksp-cadherin promoter [23]. In addition, we observed an increase of *Mitf-A* expression in S-bodies and ureteric tips (**Fig 5C**). A further increase in *Mitf-A* expression in transgenic metanephroi was found at E15.5 (**S6 Fig**). Immunohistochemical analysis corroborated these observations. In fact, it showed that endogenous MITF-A protein was expressed in both UB stalks and tips and to a much lower extent in mesenchyme (**Fig 5D**). Moreover, we observed a marked increase of MITF-A staining in UB stalks, tips and S-bodies of MITF-A transgenic embryos (**Fig 5D**). Since MITF is composed of several distinct isoforms, it was important to analyze the pattern of expression of all isoforms during nephrogenesis. Quantitative RT-PCR showed that, amongst the nine MITF isoforms, only *Mitf-A*, *Mitf-H*, *Mitf-C*, *Mitf-J* and Mitf-Mc, but not *Mitf-M*, *Mitf-B*, *Mitf-D* and *Mitf-E*, were detectably expressed in kidneys of E13.5 embryos, (**S7A Fig**). Interestingly, MITF-A overexpression was associated with an increase in *Mitf-C* and *Mitf-J* mRNA expression (**S7B Fig**), suggesting positive regulation by MITF-A.

### MITF-A affects cell proliferation and apoptosis during kidney development

**Fig 6:**
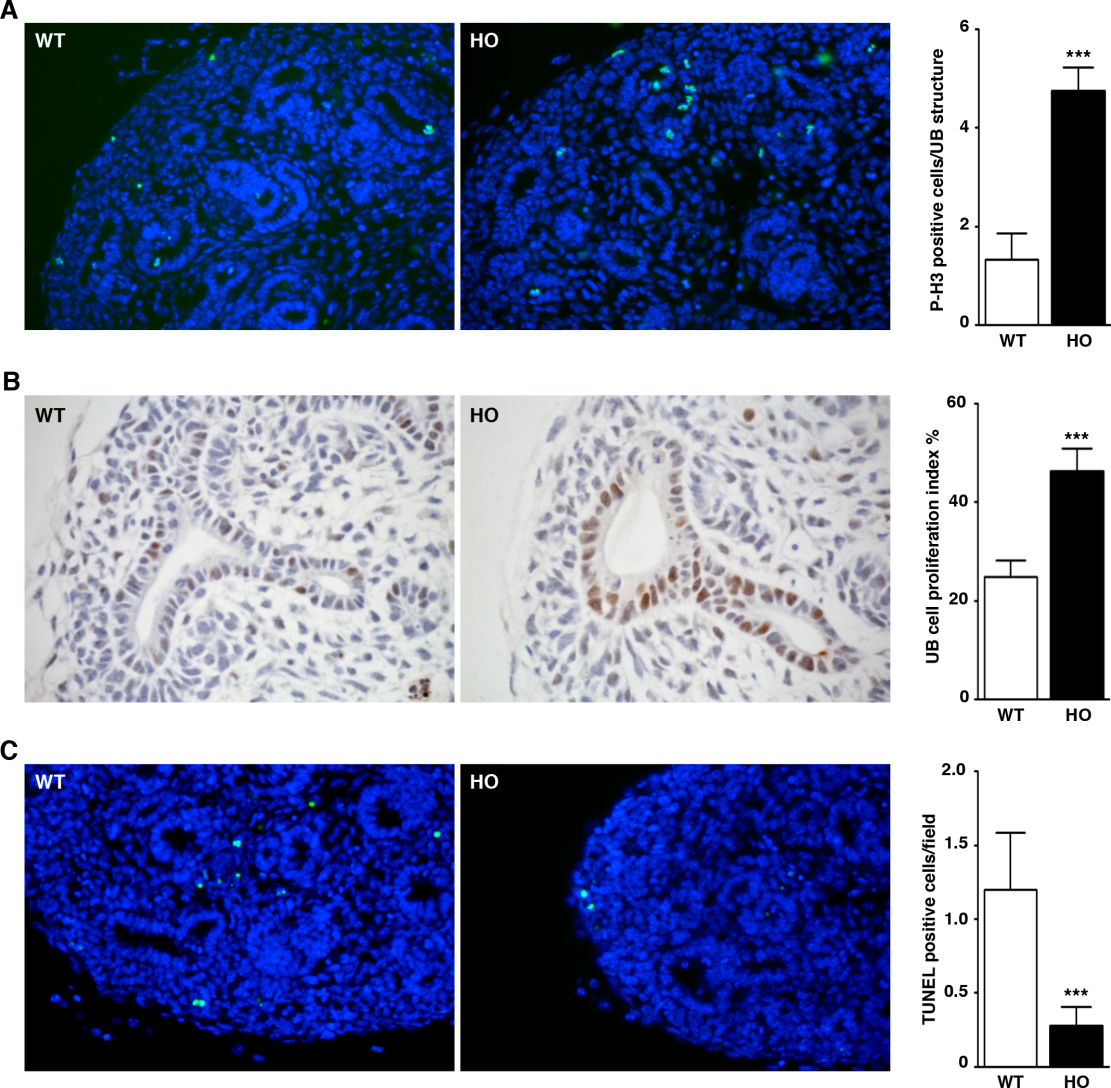
Impact of MITF-A overexpression on cell survival. **A-B)** Cell proliferation in E13.5 kidneys from wild-type (WT) and homozygous (HO) MITF-A transgenic embryos. Proliferating cells were identified using an anti-phospho-histone H3 (pH3) **(A)** and an anti-PCNA antibody **(B)**. Magnifications are X400 and X600, respectively. Left panels: representative images of 5 kidneys; right panels: quantification of the number of pH3-positive and PCNA-positive cells per UB structure. **C)** Apoptosis was evaluated by TUNEL assay in E13.5 kidneys from WT and HO MITF-A transgenic embryos. Left panels: representative images of 5 kidneys (magnification X400); right panels: quantification of the number of TUNEL-positive cells per microscopic field. Data are means ± SEM. Quantifications were performed on three sections for each kidney (n = 5 mice per genotype). Mann-Whitney test; transgenic *versus* wild-type mice: *** *P* < 0.001.

It has been well documented that both cell proliferation [7,24] and apoptosis [25] play a role in kidney development. Interestingly, MITF has been implicated in the control of both of these events [20] and so we evaluated them in our experimental model. Using phospho-histone-H3 staining, we observed a strong increase in cell proliferation in UB of E13.5 MITF-A transgenic kidneys as compared to their age-matched wild-type counterparts (**Fig 6A**). These results were confirmed using an antibody directed against PCNA, a protein selectively expressed in proliferative S phase cells (**Fig 6B**). Conversely, apoptosis, as judged by TUNEL assay, was dramatically decreased in transgenic mice compared to wild-type mice. This was, however, mostly seen in the metanephric mesenchyme and thus suggested a non cell-autonomous effect of MITF-A (**Fig 6C**).

### Identification of the potential MITF-A targets in branching morphogenesis

**Fig 7:**
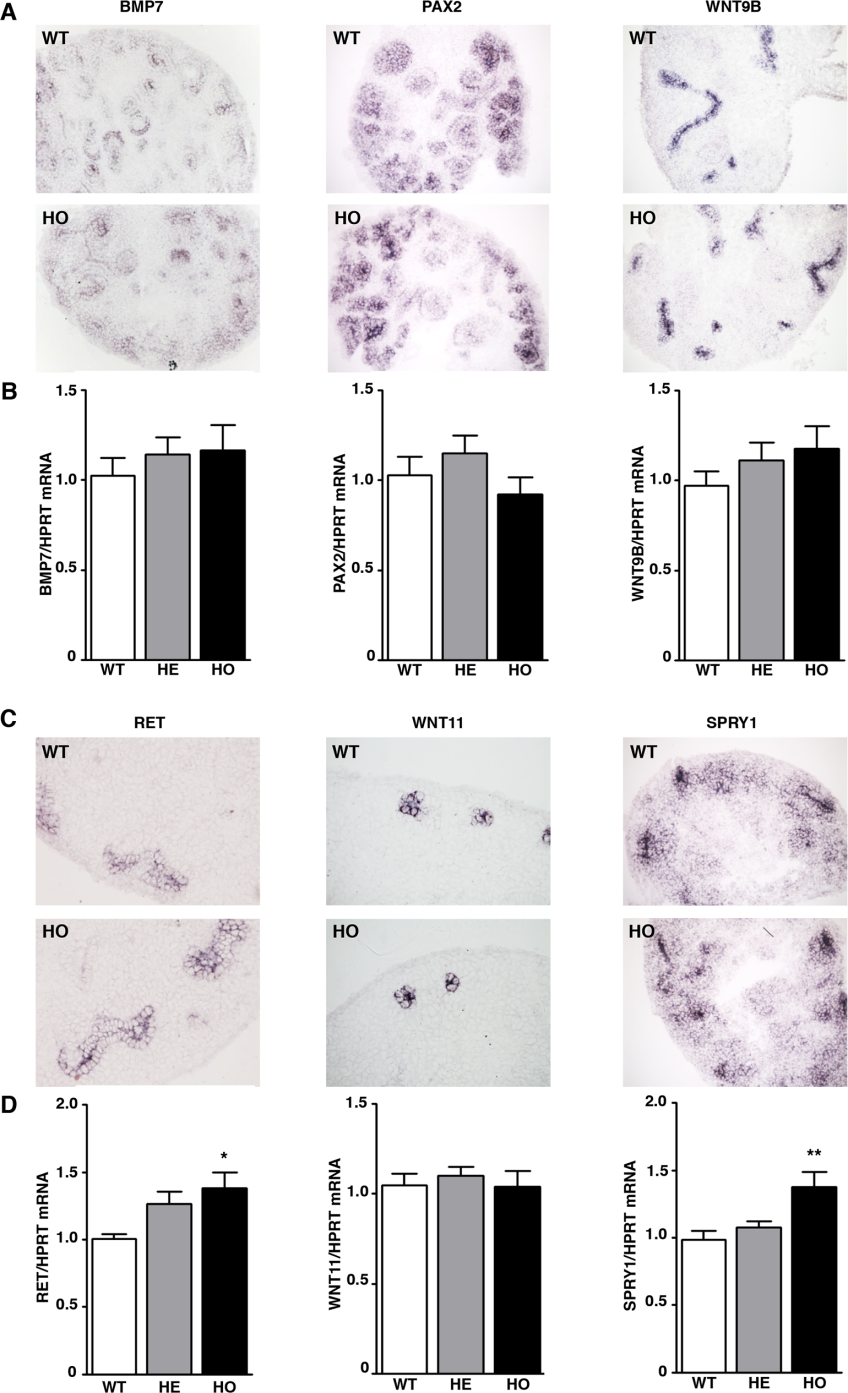
Expression of candidate MITF-A targets in E13.5 kidneys. **A)** *In situ* hybridization of *Bmp7*, *Pax2* and *Wnt9b* in wild-type (WT) and homozygous (HO) MITF-A transgenic kidneys at E13.5 (magnification X200, n = 5–6 per genotype). **B)** Quantitative RT-PCR analysis of *Bmp7*, *Pax2* and *Wnt9b* mRNA expression in E13.5 kidneys of WT, heterozygous (HE) and HO MITF-A transgenic embryos (n = 6–9 per genotype). **C)** *In situ* hybridization of *Re*t, *Wnt11* and *Spry1* in WT and HO MITF-A transgenic kidneys at E13.5 (magnification X200, n = 5–6 per genotype). Note the increased staining of *Re*t mRNA in transgenic kidneys at E13.5. **D)** Quantitative RT-PCR analysis of *Re*t, *Wnt11* and *Spry1* mRNA expression in E13.5 kidneys of WT, HE and HO MITF-A transgenic embryos (n = 6–9 per genotype). Data are means ± SEM. ANOVA followed by Tukey-Kramer test; transgenic *versus* wild-type mice: * P < 0.05, ** P < 0.01.

Since MITF-A is a transcription factor, the observed effects are likely due to changes in the expression of one or more renal developmental genes potentially under the control of MITF-A. To identify potential target genes of MITF-A during kidney development, we took advantage of a method that we recently developed to predict STAT3 functional binding sites through comparative genomics [26] and that we adapted to MITF on the basis of the 47 known genomic MITF binding sites [27]. Using this approach, we limited the analysis to genes known to be involved in kidney development (MGI abnormal kidney development data base). Of the 102 genes analyzed, we identified 81 with a slight enrichment of conserved binding sites (CBS) for MITF (**S2 Table**). By comparison, the percentage of genes showing enrichment of MITF-A CBS was lower among those specifically affecting liver development. To refine our analysis, we systematically retrieved data from the GUDMAP database (http://www.gudmap.org/) to select genes that are expressed in UB stalks and tips where the MITF-A transgene was expressed. For genes non-included in the GUDMAP database, we scanned the literature for UB localization. These analyses identified 28 potential MITF-A targets expressed in UB (**S2 Table**). Among these, we first focused our attention on *Bmp7* [28] and *Pax2* [29], both of which are known to be involved in branching morphogenesis and to cooperate with MITF in other contexts [30,31]. However, *in situ* hybridization and quantitative RT-PCR revealed that the expression of neither *Bmp7* nor *Pax2* was modified by MITF-A overexpression (**Fig 7A and 7B**). Similarly, the expression of *Wnt9b*, another potential MITF-A target which acts as a paracrine factor in the metanephric mesenchyme induction and in UB branching [32], was unaffected by MITF-A overexpression (**Fig 7A and 7B**). Likewise, RARα, a receptor for retinoic acid, the active form of vitamin A, did not show any difference. This was in a way surprising since Vitamin A deficiency during pregnancy can lead to reduced nephron number [9] similar to what we here report for *Mitfa* deficiency in mice. Hence, we next turned our attention to our MITF-A enriched genes that are known to be critically involved in branching morphogenesis, i.e. *Ret*, *Wnt11* and *Spry1* [33–35]. Interestingly, while *Wnt11* expression was unchanged in MITF-A transgenic metanephroi at E13.5, the expression of *Ret* was significantly increased (**Fig 7C and 7D**). In particular, *in situ* hybridization revealed that *Ret* mRNA expression was significantly enhanced in ureteric tips of MITF-A transgenic kidneys as compared to kidneys of wild-type littermates (**Fig 7C**). Quantitative RT-PCR showed that the increase of *Ret* mRNA paralleled MITF-A expression levels when comparing kidneys of wild-type, heterozygous and homozygous transgenic embryos (**Fig 7D**). Conversely, we observed that the expression of *Ret* was decreased in *Mitfa*^*-/-*^ embryos as compared to wild-type littermates (**S8 Fig**). Consistent with the increase of *Ret*, the expression of *Spry1*, a downstream target of *Ret*, was also found increased in kidneys of MITF-A transgenic embryos as compared to wild-type controls (**Fig 7D**). To corroborate this observation, we decided to measure the expression of other known targets of RET [36,37]. Interestingly, our results showed that several of these targets, i.e. the transcriptional regulator *Etv5*, the chemoreceptor *Cxcr4*, and the transcriptional factor *Myb* were significantly up-regulated in MITF-A transgenic embryos as compared to controls at E13.5 (**S9 Fig**). Altogether these results suggest that MITF-A might promote increased nephron endowment by modulating RET signaling.

### *Ret* heterozygosis prevents increased UB branching in MITF-A transgenic mice

**Fig 8:**
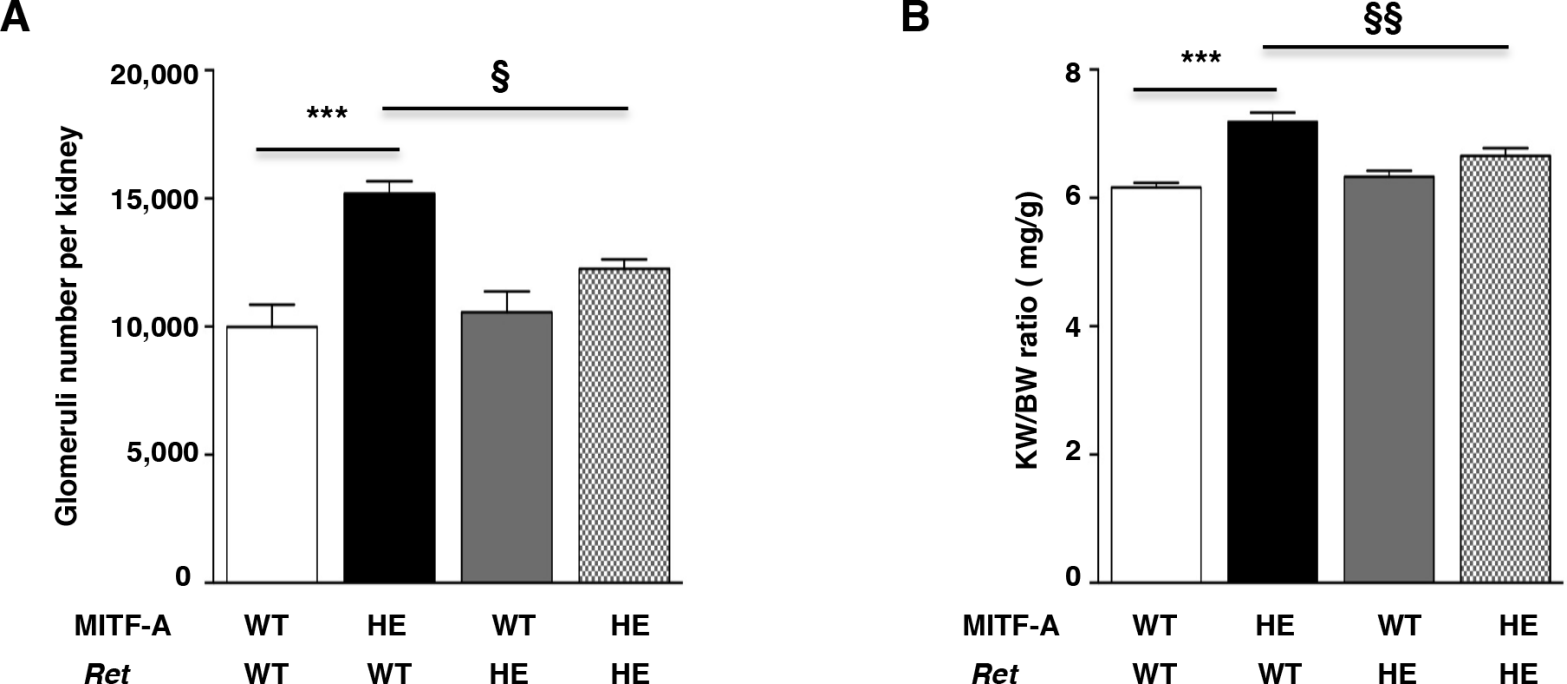
RET heterozygosis reverts MITF-A-induced phenotype. **A-B)** Glomeruli number per kidney **(A**) and kidney weight/body weight ratio (KW/BW) (**B)** in double transgenic mice generated by crossing mice overexpression MITF-A with heterozygous *Ret* knockout mice. Four groups of mice were studied: double wild-type mice (WT), heterozygous (HE) mice bearing an allele of *Ret*, HE mice overexpressing MITF-A and double HE MITF-A and *Ret* transgenic mice. Data are means ± SEM, n = 3–8 per each genotype. ANOVA followed by Tukey-Kramer test; transgenic versus wild-type mice: *** P < 0.001; MITF-A^wt/tgMITF-A^ mice *versus* MITF-A^wt/tgMITF-A^;*Ret*^*+/-*^ mice: § P < 0.05, §§ P < 0.01.

Finally, to investigate if RET is a critical effector of MITF-A, we crossed transgenic mice overexpressing MITF-A (MITF-A^wt/tgMITF-A^) with heterozygous *Ret* knockout mice (*Ret*^*wt/-*^) [38]. Remarkably, the results strongly support the genetic interaction between *Ret* and *Mitfa*. In fact, in basal conditions the heterozygosis for *Ret*, as expected, did not interfere with nephron endowment in absence of MITF-A overexpression. However, in mice overexpressing MITF-A, the heterozygosis for *Ret* dramatically reduced nephron endowment (**Fig 8A**). Consistently, the kidney weight was also significantly decreased in the transgenic MITF-A mice lacking one copy of *Ret* (**Fig 8B**). Remarkably, both the kidney weight and the number of glomeruli were similar between MITF-A^wt/tgMITF-A^*;Ret*^*wt/-*^ mice and double wild-type mice (**Fig 8**). Collectively, these results indicate that RET is the key target of MITF-A in branching morphogenesis.

## Discussion

During kidney development, ureteric bud branching morphogenesis is a fundamental process that defines the normal architecture of the kidney and the final nephron number. However, the molecular networks that orchestrate this complex biological process have been only partially elucidated. In particular, the genetic programs that regulate nephron endowment in normal kidneys are still unknown. By generating lines of transgenic and knockout mice that express different levels of MITF-A, we have uncovered an important role for this transcription factor in controlling branching morphogenesis during kidney development. Notably, we showed that MITF-A overexpression increased UB branching, which led to a substantial increase of nephron number. Conversely, *Mitfa* deficiency resulted in reduced branching and nephron number. Mechanistically, MITF-A triggered UB cell proliferation, while it inhibited mesenchyme metanephric apoptosis. In addition, by coupling an *in silico* analysis with molecular studies, we showed that amongst the putative MITF-A target genes, *Ret*, a key factor of branching morphogenesis, and its downstream signaling pathway were significantly modified by MITF-A. Consistent with the idea that *Ret* is a critical target, we showed that *Ret* heterozygosis reverted the MITF-A induced phenotype. Collectively, these results provide novel insights into the genetic networks that control branching morphogenesis in kidney development and identified one of the first modifiers of physiological nephron endowment.

Nephrogenesis starts when the metanephric mesenchyme induces the nearby Wolffian duct to produce UB outgrowth, which then elongates, invades the mesenchyme, and undergoes a process of branching morphogenesis to give rise to the renal collecting duct system. At the same time, in a reciprocal inductive process, the ureteric tips induce surrounding mesenchyme to condense, epithelialize, and differentiate into mature nephrons, the functional unit of the kidney [5]. Abnormalities of this highly coordinated morphogenetic process can lead to defects ranging from severe congenital abnormalities of kidney and urinary tract (CAKUT) [39,40] to reduced nephron number [6]. Despite the fact that the morphological events leading to nephron formation are well characterized, the genes and genetic networks that orchestrate these events are still poorly elucidated. Our study identified *Mitfa* as a novel candidate modifier of nephron endowment. To our knowledge, this is the first gene whose dosage affects exclusively the final number of nephrons without affecting overall renal morphology or function. Previous studies have shown that both the overexpression of a dominant negative isoform of the type II activin receptor [41] or *Tgfβ2* haploinsufficiency [42] lead to higher nephron number. Intriguingly, however, *Tgfβ2* homozygous mice display renal agenesis [43], whereas mice lacking activin B die at birth [44]. Similarly, while common hypomorphic variants of *RET* or *PAX2* have been associated with subtle renal hypoplasia [11,12], their inactivation has been shown to result in severe renal malformations [40]. Therefore, is seems that in contrast to *Mitfa*, these other genes have a more complex function in kidney morphogenesis and cannot be considered simply as modifiers of nephron number.

Intriguingly, we observed that in *Mitfa*^-/-^ mice, *Mitfa* deficiency affected more the final number of nephrons than the number of UB at E13.5. Since nephrogenesis is a continuous process, it is tempting to speculate that the increased nephron number may result from a mild but continuous effect of MITF-A on UB branching and nephrogenesis. Indeed, we cannot formally exclude that the overexpression of MITF-A in UB might lead to the recruitment of more mesenchymal kidney cells.

MITF-A belongs to a family of transcription factors containing seven distinct isoforms differing at their amino termini [16] among which five (MITF-A, MITF-H, MITF-C, MITF-J and MITF-Mc) we showed to be expressed in E13.5 metanephroi and in adult kidney. These isoforms, which arise from the utilization of different promoters, may differ in expression patterns but share the main functional domains, including the dimerization and DNA-binding bHLH-Zip domain [15]. Mutations in the bHLH and basic domain of *MITF* have been reported in patients with Waardenburg and Tietz syndromes [45,46]. In both syndromes, melanocyte function is severely impaired resulting in deafness, lack of pigmentation and eye abnormalities. It might be surprising that no gross abnormalities in other tissues, including kidney, have been reported in these syndromes, despite the fact that the mutations affect the common functional sequence of all *MITF* isoforms. Whether, however, these patients have a reduced nephron number is an interesting hypothesis that has not been investigated.

We previously showed that a hypomorphic variant of *Mitfa* predisposes FVB/N mice to CKD progression in an experimental model of nephron reduction [21]. In this context, we observed that MITF-A acts by interacting with histone deacetylases to repress the transcription of *Tgfa*, a ligand of EGFR and a critical mediator of CKD progression. Remarkably, the number of nephrons is identical in *Tgfa*^*-/-*^ and *Tgfa*^*+/+*^ mice (12,056 ± 561 and 11,250 ± 328, respectively), suggesting that a different genetic program is triggered by MITF-A during branching morphogenesis. To start to identify this program we combined comparative genomics with mRNA transcripts quantification. The results showed that several of the potential targets are normally expressed in MITF-A transgenic embryos. However, the expression of *Ret*, a tyrosine kinase receptor critically involved in renal branching morphogenesis [36], was significantly increased in MITF-A transgenic mice. In addition, we observed that *Ret* overexpression resulted in the activation of its signaling pathway, as judged by the up-regulation of several downstream critical targets, i.e. *Etv5*, *Spry1*, *Cxcr4* or *Myb* [36,37]. More importantly, we confirmed the genetic interaction between *Mitfa* and *Ret* and showed that *Ret* heterozygosis prevents the effect of MITF-A overexpression on nephron endowment. In fact, the number of nephrons was similar between MITF-A^wt/tgMITF-A^*;Ret*^*wt/-*^ mice and wild-type littermates. Interestingly, although *Ret* inactivation has been shown to induce a very severe kidney phenotype (renal agenesis) [40], a common single polymorphism within an exonic splicing enhancer was associated with reduced kidney size at birth [12], supporting the idea that subtle changes in *RET* expression levels might account for nephron number variability. Hence, all these data together point to *Ret* as the critical target of MITF-A during branching morphogenesis. Although we cannot ascertain that *Ret* is a direct target of MITF-A, several lines of evidence support this idea. First, our *in silico* analysis revealed that the *Ret* regulatory region contains 12 MITF binding sites that are conserved in at least 5 species. Second, we demonstrated that MITF-A is expressed in the same structures than RET, i.e. the ureteric tips, and that RET expression levels in transgenic ureteric tips increased proportionally to those of MITF-A. Third, we observed that increased UB branching in E13.5 MITF-A metanephroi was associated with a marked increase of UB cell proliferation, an event known to participate in branching morphogenesis [24] and to be modulated by ERK1/2 [47] and PI3K [48], two effectors of RET. Whether other targets of MITF-A are involved in MITF-A promoting nephrogenesis is a possibility that we cannot formally exclude.

It has been previously shown that the overexpression of the anti-apoptotic protein BCL2 in the developing kidneys of *Pax2*^*+/-*^ mice prevents the decrease of UB branching and nephron formation by suppressing cell apoptosis [49]. In addition, in the same model, it has been observed that BCL2 overexpression alone leads to increased kidney weight and increased nephron number. Conversely, *Bcl2* gene inactivation results in decreased UB branching and renal hypoplasia, but also in severe cystic dysplasia [50]. Our study showed that MITF-A overexpression in UB lead to reduced apoptosis, but mainly in metanephric mesenchyme. Interestingly, BCL2 has been shown to be a direct target of MITF-M in melanocytes [51]. However, previous studies showed that BCL2 is exclusively expressed in metanephric mesenchyme, indicating that the inhibition of apoptosis in MITF-A transgenic embryos is mainly non-cell autonomous and, therefore, not involve BCL2 as a direct target.

In conclusion, our study has revealed a novel function of MITF-A and highlighted its crucial role in kidney morphogenesis and nephron endowment. Hence, it is conceivable that polymorphisms in the *MITF* gene might influence inter-individual differences in nephron number that one can observe in the human population. The fact that suboptimal nephron number has been shown to predispose people to hypertension and CKD points to MITF-A as a potential prognostic marker for identifying patients at risk of renal disease.

## Supporting information

S1 Table Renal morphometry in wild-type and MITF-A transgenic mice. WT: wild-type mice; HO: homozygous MITF-A transgenic mice. Measures were performed in kidneys from 2 month-old mice of line 42. Data are means ± SEM; n = 4–6 per each genotype. Mann-Whitney test; HO v*ersus* WT mice: ^a^ P *<* 0.05.
(PDF)

S2 Table MITF-A potential targets involved in kidney development. Column 2 indicates the number of MITF-A putative conserved binding sites (CBS) within 30 kb, 10 kb, 5 kb and 2 kb genomic sequence upstream the transcription starting site.
Column 3 indicates the protein name of each gene.
Column 4 indicates the expression site for each gene according to either Gudmap data or published studies. na: not available; UB: ureteric bud; MM: metanephric mesenchyme; SB: S-shaped bodies; CB: C-shaped bodies; CM: condensed mesenchyme. The 28 genes expressed in UB appear in bold.
(PDF)

S3 Table PCR and RT-qPCR primer sequences. (PDF)

S1 Fig The transgene MITF-A is selectively expressed in kidney. Transgene (tg) *MITF-A* mRNA expression evaluated by quantitative RT-PCR in kidney, liver, spleen, heart, lung and brain from MITF-A transgenic mice of line 42, 2 months after birth. Data are means ± SEM; n = 4 per organ.
(TIF)

S2 Fig The MITF-A induced phenotype is confirmed in different transgenic lines. **A, D)** *Mitf-A* mRNA expression evaluated by quantitative RT-PCR in kidneys from wild-type (WT) and heterozygous (HE) MITF-A transgenic mice from line 14 (**A**) and 47 (**D**), 2 months after birth. **B, E)** Glomeruli number per kidney in WT and HE MITF-A transgenic females and males of line 14 (**B**) and 47 (**E**), 2 months after birth. **C, F)** Kidney morphology from WT and HE MITF-A transgenic mice from line 14 (**C**) and 47 (**F**), 2 months after birth. PAS staining; magnification: X200. Because morphology of transgenic mice and wild type mice were indistinguishable between females and males, only data of female mice are shown. Data are means ± SEM; n = 4–5 per genotype and sex. Mann Whitney test; transgenic *versus* wild-type mice: * P < 0.05, *** P < 0.001.
(TIF)

S3 Fig MITF-A overexpression increases kidney weight. Kidney weight/body weight (KW/BW) ratio from wild-type (circles) and heterozygous MITF-A transgenic (square symbol) mice (line 42) at birth (W0) and 3 (W3), 8 (W8), 16 (W16), and 24 (W24) weeks after birth. Because KW/BW data obtained from wild-type and transgenic mice showed the same profile in females and males, only data from females are shown. Data are means ± SEM (n = 3–7 per group). Mann Whitney test; transgenic *versus* wild-type: * P < 0.05, ** P < 0.01.
(TIF)

S4 Fig MITF-A overexpression does not affect renal morphology and function. **A**) Kidney morphology from wild-type (WT), heterozygous (HE) and homozygous (HO) MITF-A transgenic mice (line 42) at 2, 4, 6 and 12 months after birth. PAS staining; magnification: X200. **B)** Plasma urea levels (left panel), urinary albumin/creatinine ratio (middle panel) and urinary protein/creatinine ratio (right panel) in WT, HE and HO MITF-A transgenic mice at 12 months. Because morphological and biological data from transgenic mice and wild type mice were indistinguishable between females and males, only data of female mice are shown. Any statistically significant difference was observed among the three experimental groups.
(TIF)

S5 Fig UB number in embryonic kidneys. **A)** Ureteric bud (UB) branching, as assayed by counting the number of UB tips in wild-type (WT, n = 17), heterozygous (HE, n = 14) and homozygous (HO, n = 25) MITF-A transgenic metanephroi at E13.5. Data are means ± SEM. ANOVA followed by Tukey-Kramer test; transgenic *versus* wild-type mice: *** P < 0.001, HE v*ersus* HO MITF-A transgenic mice: ## P < 0.01. **B)** Number of UB tips in E13.5 kidneys from *Mitfa*^*+/+*^ (n = 15) and *Mitfa*^*-/-*^ (n = 20) embryos. Data are means ± SEM. Mann-Whitney test; *Mitfa*^*-/-*^ versus: *Mitfa*^*+/+*^: * P < 0.05.
(TIF)

S6 Fig MITF-A expression pattern in kidneys. *In situ* hybridization of *Mitf-A* in kidneys of wild type (WT) and homozygous (HO) MITF-A transgenic embryos at E15.5. Note the strong staining in UB and early tubules in MITF-A transgenic kidneys. Magnifications: X100 (left panels) and X400 (right panels), n = 5–6 per genotype.
(TIF)

S7 Fig mRNA expression levels of *Mitf* isoforms. **A**) *Mitf* isoform expression levels in kidneys of wild-type embryos at E13.5 as judged by Ct values obtained from RT-PCR curves (Ct values inversely correlate with mRNA levels). Note that the other isoforms, namely *Mitf-M*, *Mitf-B*, *Mitf-D* and *Mitf*-*E* were undetectable in kidneys. **B**) *Mitf-H*, *Mitf-C*, *Mitf-J* and *Mitf-Mc* expression levels in kidneys of wild-type (WT) and homozygous (HO) transgenic embryos at E13.5. Data are means ± SEM; n = 5–6 per genotype. Mann Whitney test; transgenic *versus* wild-type mice: * P < 0.05.
(TIF)

S8 Fig *Ret* mRNA expression levels in *Mitfa* null mice. *Ret* mRNA expression evaluated by quantitative RT-PCR in kidneys from *Mitfa*^*+/+*^ and *Mitfa*^*-/-*^ embryos at E13.5. Data are means ± SEM; n = 3–5 per genotype. Mann Whitney test; *Mitfa*^*+/+*^ versus *Mitfa*^*-/-*^ mice: * P < 0.05.
(TIF)

S9 Fig RET signaling is activated upon MITF-A overexpression. mRNA expression of known RET targets evaluated by quantitative RT-PCR in kidneys from wild-type (WT), heterozygous (HE) and homozygous (HO) MITF-A transgenic embryos at E13.5. Data are means ± SEM; n = 7–8 per genotype. ANOVA followed by Tukey-Kramer test; transgenic *versus* wild-type: ** P < 0.01.
(TIF)
