# Supplementary material for: MITF – A controls branching morphogenesis and nephron endowment
Source: PLoS Genet. 2017 Dec 14;13(12):e1007093. doi: 10.1371/journal.pgen.1007093 (PMC5746285; doi:10.1371/journal.pgen.1007093)
Supplement: S1 Table — WT: wild-type mice; HO: homozygous MITF-A transgenic mice. Measures were performed in kidneys from 2 month-old mice of line 42. Data are means ± SEM; n = 4–6 per each genotype. Mann-Whitney test; HO versus WT mice: a P < 0.05. (PDF) [file pgen.1007093.s001.pdf]

S1 Table: Renal morphometry in wild-type and MITF-A transgenic mice.

	Glomerular surface (μm^2)	Tubular cell surface (μm^2)	Kidney weight (mg)	Glomeruli number	Kidney weight /glomeruli number (%)
Females					
WT	2169 ± 62	469 ± 24	126 ± 7	13,726 ± 519	0.92 ± 0.04
HO	1843 ± 70[a]	491 ± 8	158 ± 3[a]	17,983 ± 318[a]	0.88 ± 0.01
Males					
WT	2300 ± 113	653 ± 36	196 ± 9	19,938 ± 419	0.98 ± 0.05
HO	1900 ± 47[a]	603 ± 10	238 ± 9[a]	25,385 ± 528[a]	0.94 ± 0.04
