# Supplementary material for: MITF – A controls branching morphogenesis and nephron endowment
Source: PLoS Genet. 2017 Dec 14;13(12):e1007093. doi: 10.1371/journal.pgen.1007093 (PMC5746285; doi:10.1371/journal.pgen.1007093)
Supplement: S2 Table — Column 2 indicates the number of MITF-A putative conserved binding sites (CBS) within 30 kb, 10 kb, 5 kb and 2 kb genomic sequence upstream the transcription starting site. Column 3 indicates the protein name of each gene. Column 4 indicates the expression site for each gene according to either Gudmap data or published studies. na: not available; UB: ureteric bud; MM: metanephric mesenchyme; SB: S-shaped bodies; CB: C-shaped bodies; CM: condensed mesenchyme. The 28 genes expressed in UB appear in bold. (PDF) [file pgen.1007093.s002.pdf]

Appendix Table S2: MITF-A potential targets involved in kidney development.

Gene Symbol	CBS (30, 10, 5, 2 kb)	Name	localization	Reference
Amrh2	11, 4, 3, 3	Anti-mullerian hormone type-2 receptor	na	na
Arl3	1, 1, 1, 0	ADP-ribosylation factor-like protein 3	na	na
Axin1	5, 2, 1,0	Axin-1 (Axis inhibition protein 1)	na	na
Bag6/Bat3	35, 13, 4, 1	HLA -B associated transcript 3 (Bat 3 protein)	na	1
Bcl2	17, 11, 9, 9	Apoptosis regulator B cell lymphoma 2 (Bcl2 protein)	CM	Gudmap
BMP4	4, 4, 2, 1	Bone morphogenetic protein 4	MM	Gudmap
**Bmp7**	**18, 12, 8, 5**	**Bone morphogenetic protein 7**	**UB**	**Gudmap**
Cdh6	7, 2, 2, 1	Cadherin 6, K-Cadherin	renal vesicle	Gudmap
Cbs	7, 1, 1, 0	Cystathionine beta-synthase	na	na
Cdkn1c	2, 1, 1, 0	Cyclin-dependent kinase inhibitor 1C (P57 Kip2 protein)	renal vesicle	Gudmap
Cited2	5,5, 5, 3	Cbp/p300-interacting transactivator 2	early tubule	Gudmap
Ctnnb1	14, 3, 3, 0	Catenin beta -1	early tubule	Gudmap
Ctnnbip1	1,0, 0, 0	Beta catenin interacting protein	CM	Gudmap
Ctnnd1	8, 1, 1, 1	P120 catenin (P120 ctn)	early tubule	Gudmap
Dchs 1	29, 17, 11, 5	Protein Dchs 1	MM	2
**Emx2**	**10, 5, 2, 0**	**Homeobox empty spiracles-like homolog 2 (Emx2 protein)**	**UB tip**	**Gudmap**
Eya1	12, 10, 5, 0	Eyes absent homolog 1	CM	Gudmap
Fat4	13, 10, 9, 2	Protocadherin Fat4	MM	2
Fgf10	16, 12, 10 8	Fibroblast growth factor 10	MM	3
Fgf7	3, 3, 3, 2	Fibroblast growth factor 7	MM	4
Fgf8	25, 13, 8, 5	Fibroblastst growth factor	renal vesicles	5
Fgfr1	9, 5, 5, 2	Fibroblast growth factor receptor 1	CM	Gudmap
**Fgfr2**	**10, 5, 4, 0**	**Fibroblast growth factor receptor 2**	**UB tip and trunk**	**Gudmap**
Fgfrl1/fgfr5	14, 2, 1, 1	Fibroblast growth factor receptor-like 1	MM	6
Frem1	1,1,1, 1	Fras1 related extracellular matrix protein 1 (Frem 1 protein)	na	na
**Fzd4**	**21,14, 14, 6**	**Frizzled-4 protein**	**UB tip**	**Gudmap**
**Fzd8**	**9,9, 9, 6**	**Frizzled-8 protein**	**UB, UB tip**	**7**
Galc	4, 1, 0, 0	Galactosylcerebrosidase/galactoceremidase	na	na
**Gdf11/Bmp11**	**9, 5, 0, 0**	**Growth differentiation factor 11**	**CM, ureteric branches**	**8**
Gdnf	6, 3, 3, 0	Glial line-derived neurotrophic factor	CM	9
**GFRa1**	**1, 1, 1, 0**	**GDNF family receptor alpha-1**	**UB tip and tree**	**Gudmap**
**Gpc3**	**2, 2 ,2 ,2**	**Glypican-3**	**CM, interstitium, UB tip**	**Gudmap**
Grem1	2 ,1, 0, 0	Gremlin-1	MM	10
Hoxa11	19, 6, 5, 4	Homeobox protein Hox-A11	CM	Gudmap
Hoxc11	21, 6, 8, 2	Homeobox protein Hox-C11	MM, stroma	11
Hoxd11	25, 7, 3, 2	Homeobox protein Hox-D11	MM, stroma	11
Igf2bp1	3, 2, 2, 1	Insulin-like growth factor 2 mRNA binding protein	developping tubules	12
**Ilk**	**40, 12, 4, 0**	**Integrin-linked protein kinase**	**UB, MM**	**13**
Itga8	7, 4, 1, 1	Integrin alpha 8	MM	14
JMJD6	13, 10, 1, 1	Bifunctional arginine demethylase and lysyl-hydroxylase JMJD6	na	na
**Lama5**	**8, 0, 0, 0**	**Laminin alpha 5**	**UB trunk, renal corpuscle**	**Gudmap**
Lamc1	5, 5, 5, 5	Laminin gamma 1 subunit (lamc1 protein)	SB, renal corpuscle	Gudmap
Lhx1/Lim1	14, 10, 4, 1	LIM / homeobox protein Lhx1	early tubule, early nephron	Gudmap
Lrg4	5, 1, 0, 0	Leucine-rich repeat containing protein coupled receptor 4	na	na
**Lrp4**	**22, 1, 1, 1**	**Low density lipoprotein receptor-related protein 4**	**early tubule, UB**	**Gudmap**
**Mmp14**	**10, 7, 3, 1**	**Matrix metalloproteinase-14**	**UB, MM**	**15**
**Notch2**	**3, 1, 1, 0**	**Neurogenic locus notch homolog protein 2**	**UB, renal vesicle, tubule**	**Gudmap**
**Npnt**	**2, 1, 1, 1**	**Nephronectin**	**UB, renal corpuscle**	**Gudmap**
Osr1	5, 5, 2, 0	Protein odd-skipped-related 1	CM, MM	Gudmap
Parva	3, 1, 1, 1	Parvin-alfa (Actopaxin)	renal corpuscle	Gudmap
**Pax2**	**12, 8, 5, 1**	**Paired box protein Pax-2**	**UB, CM**	**Gudmap**
**Pax8**	**4, 2, 2, 1**	**Paired box protein Pax-8**	**UB, renal vesicle, tubule**	**Gudmap**
**Pex 5**	**13, 1, 1, 1**	**Peroxisomal targeting signal 1 receptor (Peroxin-5)**	**UB, CM**	**Gudmap**
**Plxnb1**	**23, 8, 5, 1**	**Plexin-B1**	**UB, renal corpuscle**	**16**
**Ptgs2/Cox2**	**5, 5, 3, 2**	**Prostaglandin G/H synthase 2/cycloxygenase (Cox2 protein)**	**UB, MM**	**17**
**Ptrh2**	**12, 0, 0, 0**	Peptidyl tRNA hydrolase 2 (Bit1)	na	na
**Pygo2**	**18, 12, 6, 1**	**Protein pygo 2**	**UB, MM**	**18**
**Rara**	**41, 17, 10, 5**	**Retinoic acid receptor alpha (RARA)**	**CM, UB tip**	**Gudmap**
Rarg	22, 8, 5, 3	Retinoic acid receptor gamma (RARG)	intersitium, stroma	Gudmap
Rdh10	4, 4, 4, 3	Retinol deshydrogenase 10	CM	19
**Ret**	**9, 3, 0, 0**	**Proto-oncogene tyrosine-protein kinase receptor Ret**	**UB tip**	**Gudmap**
Rspo2	7, 5, 4, 2	Roof plate-specific spondin -2 (R-spondin-2)	na	na
Sall1	29, 10, 2, 2	Sal-like protein 1	CM, renal vesicle	Gudmap
Six1	20, 17, 12, 4	Homeobox protein Six1 (Sin oculis homeobox homolog 1)	CM	20
Six2	6, 1, 1, 1	Homeobox protein Six2 (Sin oculis homeobox homolog 2)	CM	Gudmap
Slc19a1	4, 2, 0, 0	Folate transporter 1	early tubule	Gudmap
Smad4	5, 0, 0, 0	Mothers against decapentaplegic protein homolog 4	CM, ueteric tip	Gudmap
**Sox4**	**4, 4, 4, 1**	**Transcription factor Sox- 4**	**UB, early tubule**	**Gudmap**
**Spy1**	**5, 4, 4, 1**	**Protein sprouty homolog 1**	**UB, MM**	**21**
Stk36	31, 14, 6, 2	Serine/threonine protein kinase 36	na	na
Tcf21	4, 4, 4, 2	Transcription factor 21 (Pod 1)	renal interstitium	Gudmap
**Trps1**	**12, 7, 5 1**	**Zinc-finger transcription factor Trps1**	**UB trunk**	**Gudmap**
Twsg1	3, 1, 1, 0	Twisted gastrulation protein homolog1	na	na
VEGFa	5, 4, 3, 2	Vascular endothelial growth factor	early tubules, renal corpuscle	Gudmap
**Wnt11**	**19, 5, 2, 1**	**Wingless-type MMTV integration site family, member 11**	**UB tip**	**Gudmap**
Wnt4	21,7,3 ,2	Wingless-type MMTV integration site family, member 4	early nepron, C-shaped body	Gudmap
**Wnt7b**	**11, 7, 3, 0**	**Wingless-type MMTV integration site family , member 7b**	**UB trunk**	**Gudmap**
**Wnt9b**	**19,0,0,0**	**Wingless-type MMTV integration site family, member 9B**	**UB trunk and tip**	**Gudmap**
WT1	2, 0, 0, 0	Wilms tumor protein homolog	CM, early tubule	Gudmap
Xdh	2, 0, 0, 0	Xanthine dehydrogenase/oxidase	na	na

1. Desmots F, et al. (2005). The reaper-binding protein scythe modulates apoptosis and proliferation during mammalian development. Mol Cell Biol 25: 10329-10337.
2. Mao ., et al. (2011). Characterization of a Dchs1 mutant mouse reveals requirements for Dchs1-Fat4 signaling during mammalian development. Development 138: 947-957.
3. Michos O, et al. (2010). Kidney development in the absence of Gdnf and Spry1 requires Fgf10. PLoS Genet 6: e1000809.
4. Qiao J, et al. (1999). FGF-7 modulates ureteric bud growth and nephron number in the developing kidney. Development 126: 547-554.
5. Grieshammer, U, et al. (2005). FGF8 is required for cell survival at distinct stages of nephrogenesis and for regulation of gene expression in nascent nephrons. Development 132: 3847-3857.
6. Gerber SD, et al. (2009). The murine Fgfrl1 receptor is essential for the development of the metanephric kidney. Dev Biol 335: 106-119.
7. Ye X, et al. (2011). Genetic mosaic analysis reveals a major role for frizzled 4 and frizzled 8 in controlling ureteric growth in the developing kidney. Development 138: 1161-1172.
8. Esquela AF, and SJ Lee (2003). Regulation of metanephric kidney development by growth/differentiation factor 11. Dev Biol 257: 356-370.
9. Sainio K, et al. (1997). Glial-cell-line-derived neurotrophic factor is required for bud initiation from ureteric epithelium. Development 124: 4077-4087.
10. Michos O, et al. (2004). Gremlin-mediated BMP antagonism induces the epithelial-mesenchymal feedback signaling controlling metanephric kidney and limb organogenesis. Development 131: 3401-3410.
11. Patterson LT, et al. (2001). Hoxa11 and Hoxd11 regulate branching morphogenesis of the ureteric bud in the developing kidney. Development 128: 2153-2161.
12. Hansen TV, et al. (2004). Dwarfism and impaired gut development in insulin-like growth factor II mRNA-binding protein 1-deficient mice. Mol Cell Biol 24: 4448-4464.
13. Smeeton J, et al. (2010). Integrin-linked kinase regulates p38 MAPK-dependent cell cycle arrest in ureteric bud development. Development 137: 3233-3243.
14. Muller U, et al. (1997). Integrin alpha8beta1 is critically important for epithelial-mesenchymal interactions during kidney morphogenesis. Cell 88: 603-613.
15. Legallicier B, et al. (2001). Expression of the type IV collagenase system during mouse kidney development and tubule segmentation. J Am Soc Nephrol 12: 2358-2369.
16. Korostylev A, et al. (2008). A functional role for semaphorin 4D/plexin B1 interactions in epithelial branching morphogenesis during organogenesis. Development 135: 3333-3343.
17. Zhang MZ, et al. (1997). Cyclooxygenase-2 in rat nephron development. Am J Physiol 273: F994-1002.
18. Schwab KR,et al. (2007). Pygo1 and Pygo2 roles in Wnt signaling in mammalian kidney development. BMC Biol 5: 15.
19. Cammas L, al. (2007). Expression of the murine retinol dehydrogenase 10 gene correlates with many sites of retinoid signalling during embryogenesis and organ differentiation. Dev Dyn 236: 2899-2908.
20. Xu PX, et al. (2003). Six1 is required for the early organogenesis of mammalian kidney. Development 130: 3085-3094.
21. Yosypiv IV, et al. (2008). Downregulation of Spry-1, an inhibitor of GDNF/Ret, causes angiotensin II-induced ureteric bud branching. Kidney Int 74: 1287-1293.
