# Supplementary material for: MITF – A controls branching morphogenesis and nephron endowment
Source: PLoS Genet. 2017 Dec 14;13(12):e1007093. doi: 10.1371/journal.pgen.1007093 (PMC5746285; doi:10.1371/journal.pgen.1007093)
Supplement: S3 Table — (PDF) [file pgen.1007093.s003.pdf]

S3 Table: PCR and RT-qPCR primer sequences.

	**Forward Primer**	**Reverse Primer**
MITF-A transgene allele	5′-CGCCAGGAGCTTCAAAAACA-3′	5′-GCTAGCACGCGTCAGCTGACTA-3′.
MITF-A null allele	5'-AGACTGCATTTTCAACAAGTCTC	5'-TATTCCAGACAGCTTGCCCAGC-3'
MITF-A wild type allele	5'-CCCAGGCGGAACCACAA-3'	5'-TCCCTCTTGCCCGCCTTC-3'
MITF construct *in situ* hybridization	5'-CCTCCCGCTCTGGTGTGAGA-3'	5'-GGTGGTGGGCACGCTGAC-3'
MITF-A cDNA	5'-CTGGTCCAAGTCCCAAGCA -3'	5'-CGCCACGATTCCCGATT-3'
MITF-C cDNA	5'-TCCCACCAGCTGATTCCTCTA-3'	5'-TGGAAGAGAGGCATCGAGTCA-3'
MITF-H cDNA	5'-GGCGCTTAGATTTGAGATG-3'	5'-GGCGTAGCAAGATGCGTCAT-3'
MITF-J cDNA	5'-ACTACTTGTCTCGCCGTGTCTC-3'	5'-TGTCTGACTCACGGCCACT-3'
MITF-Mc cDNA	5'-TTCACACAAGCCCTACCTCAGA-3'	5'-GAGCTTAACGGAGGCTTGGA-3'
MITF-B cDNA	5-TGGTGGCATTTAGGATACCC-3'	5'-TGGCGTAGCAAGATGCGTGAT-3'
MITF-D cDNA	5'-GTTGGGACCTGACAGGCTCTGA-3'	5'-TGGCGTAGCAAGATGCGTGAT-3'
MITF-E cDNA	5'-GCCCAGTGAGGTTACGTATCTT-3'	5'-AGAGAGTGGCCGTGAGTCA-3'
MITF-M cDNA	5'-GGATTGGTGCCACCTAAAACA-3'	5'-TGGGTCTGCACCTGATAG-3'
MITF total cDNA	5'-CTTTCCTTGGTCAAGCAGTACAG-3'	5'-GTGCTCATTATAGTCAAGGGCATATCC-3'
HPRT cDNA	5'-CTTTCCTTGGTCAAGCAGTACAG-3'	5'-AAGTGCTCATTATAGTCAAGGGCATATCC-3'
BMP7 cDNA	5'-ACTGACGCCGACATGGTCAT-3'	5'-TGGTATCGAGGGTGGAAGAATT-3'
PAX2 cDNA	5'-CGCTCCAACGGTGAGAAGAG-3'	5'-GTGGGCAGGATCAGTGTATACC-3'
RARα cDNA	5'-CACAGACCTTCGGAGCATCA-3'	5'-GCCTGGGATCTCCATCTTCAA-3'
RET cDNA	5'-AGAAGGCGAGTTTGGAAAAGTTG-3'	5'-CTGGGAGGCGTTTTCTTTCA-3'
SPRY1 cDNA	5-'GAGGCCGAGGATTTCAGATG-3'	5'-TGATCTCCAGTTCCAGCAGTCA-3'
WNT9b cDNA	5'-GGCCTTCCTGTATGCAGTGT-3'	5'-CCTCAGGTCCTTGCTTCCTC-3'
WNT11 cDNA	5'-GCCCGTTTCCCTGTATGTGA-3'	5'-GCCAACTAGGGTTATGTTTCAAGAC-3'
GDNF cDNA	5'-CTTGGCCTTCTGATCCTCTGA-3'	5'-CCTGTGGATACGGTGTGATTGA-3'
ETV4 cDNA	5'-AGGACCTCAGTCACTTCCAAGAGA-3'	5'-CAGGAACAAACTGCTCATCACTGT-3'

ETV5 cDNA	5’-GGCCGAGGCATGGAATTTA-3’	5’-TGGCCGATTCTTCTGGATACC-3’
CRFL1 cDNA	5’-CCGGCACCGTTTACTTCGT-3’	5’-CTCCAGATTCCCGCCTTTTT-3’
CXCR4 cDNA	5’-GGATCTTCCTGCCCACCAT-3’	5’-TGACCAGGATCACCAATCCA-3’
DUSP6 cDNA	5’-CCTGAGGCCATTTCTTTCATAGA-3’	5’-GATACCTGCCAAGCAATGCA-3’
MYB cDNA	5’-GAGACCCCGACACAGCATCTA-3’	5’-CCATCGTAGTCATGGTCACACAT-3’
