# Supplementary figures and images for: MITF – A controls branching morphogenesis and nephron endowment
Source: PLoS Genet. 2017 Dec 14;13(12):e1007093. doi: 10.1371/journal.pgen.1007093 (PMC5746285; doi:10.1371/journal.pgen.1007093)

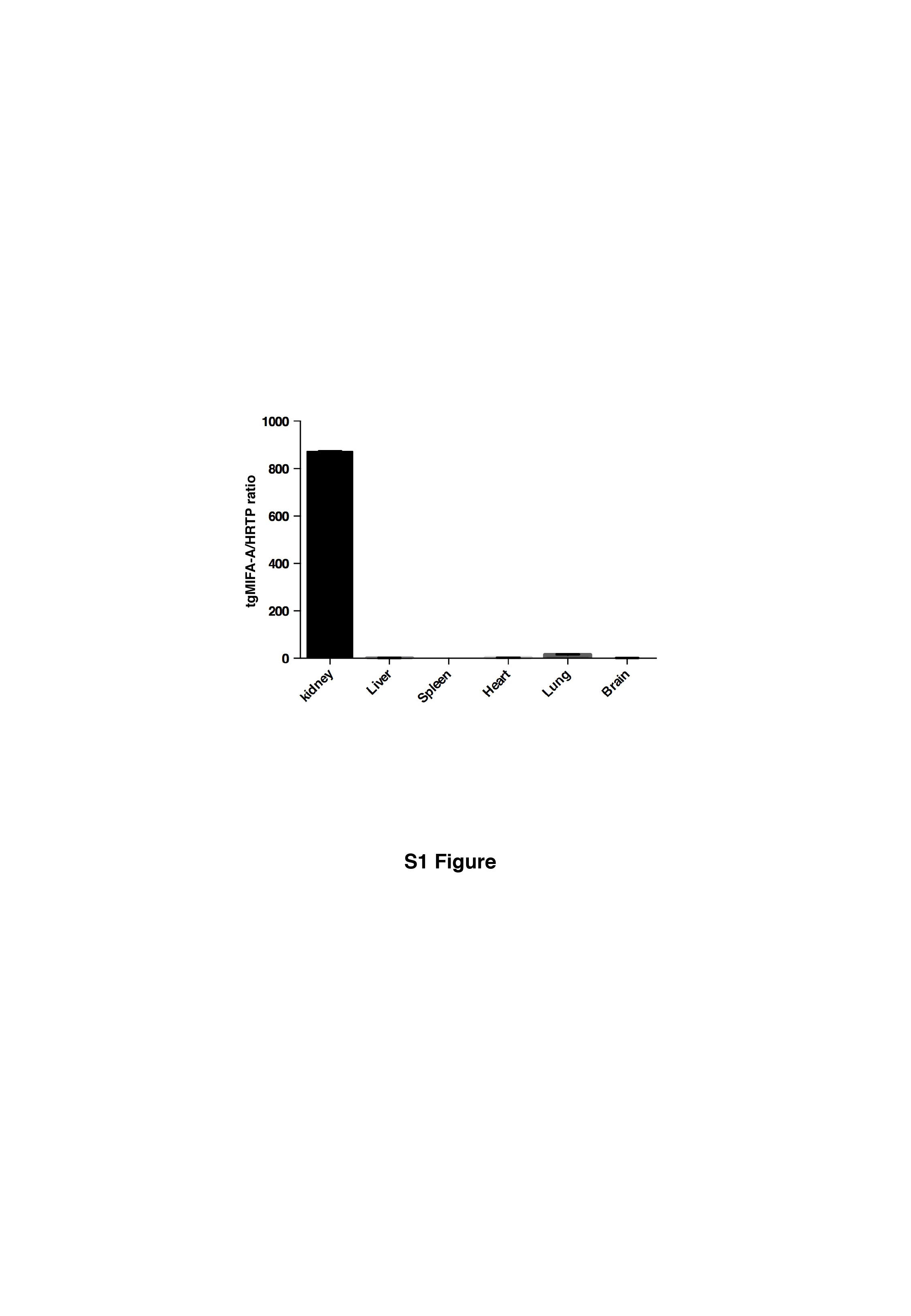

Supplement: S1 Fig — Transgene (tg) MITF-A mRNA expression evaluated by quantitative RT-PCR in kidney, liver, spleen, heart, lung and brain from MITF-A transgenic mice of line 42, 2 months after birth. Data are means ± SEM; n = 4 per organ. (TIF) [file pgen.1007093.s004.tif]

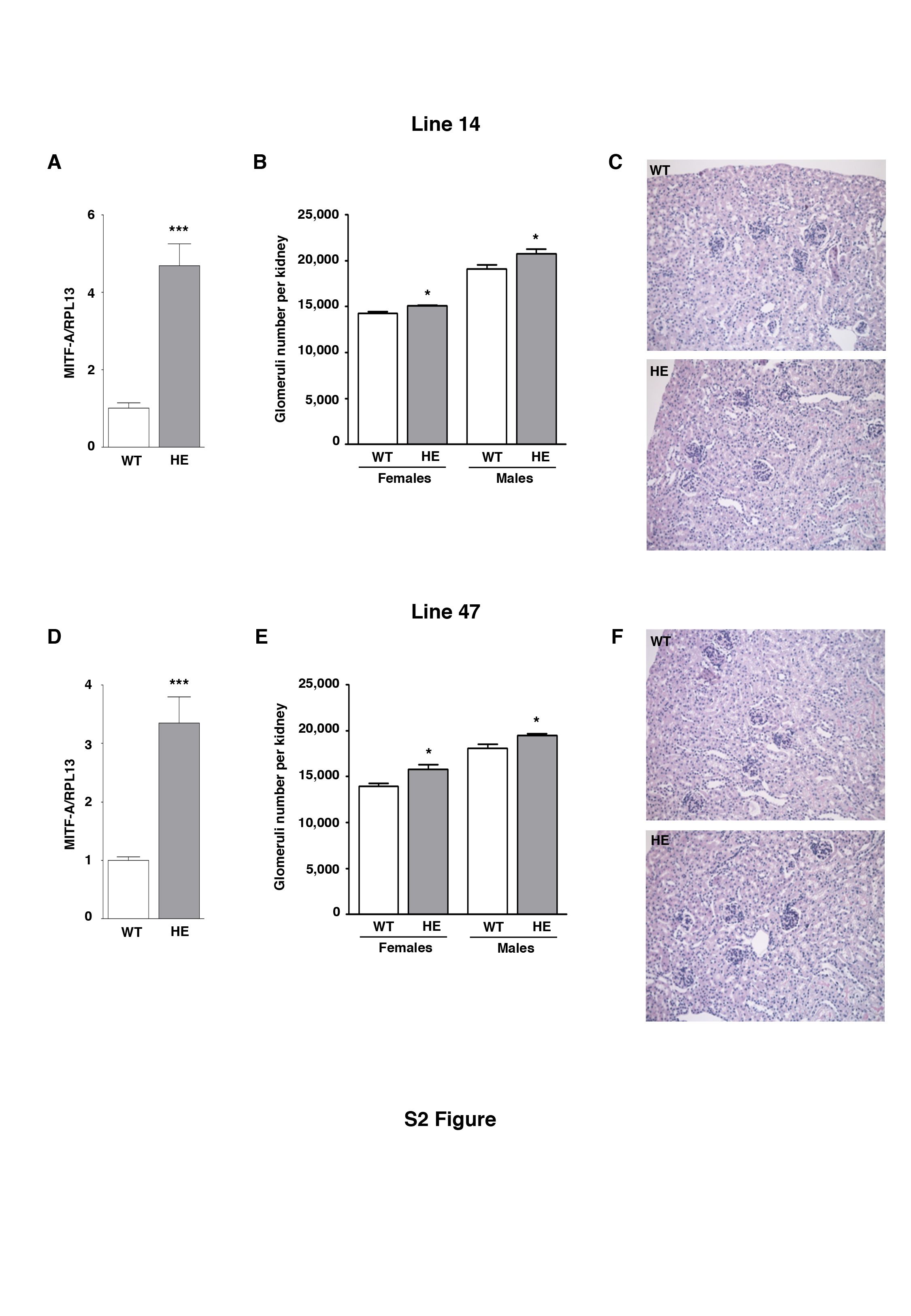

Supplement: S2 Fig — A, D) Mitf-A mRNA expression evaluated by quantitative RT-PCR in kidneys from wild-type (WT) and heterozygous (HE) MITF-A transgenic mice from line 14 (A) and 47 (D), 2 months after birth. B, E) Glomeruli number per kidney in WT and HE MITF-A transgenic females and males of line 14 (B) and 47 (E), 2 months after birth. C, F) Kidney morphology from WT and HE MITF-A transgenic mice from line 14 (C) and 47 (F), 2 months after birth. PAS staining; magnification: X200. Because morphology of transgenic mice and wild type mice were indistinguishable between females and males, only data of female mice are shown. Data are means ± SEM; n = 4–5 per genotype and sex. Mann Whitney test; transgenic versus wild-type mice: * P < 0.05, *** P < 0.001. (TIF) [file pgen.1007093.s005.tif]

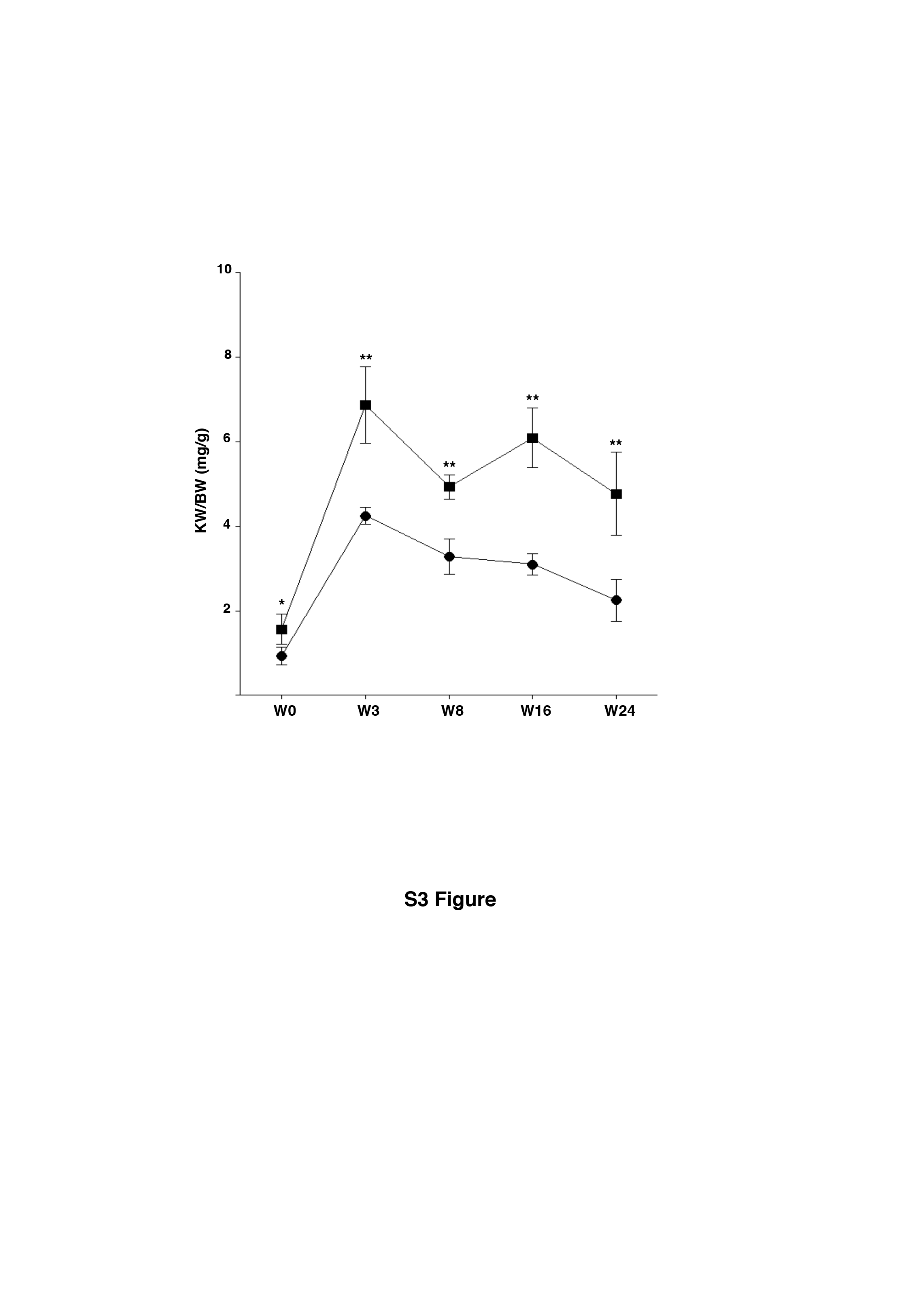

Supplement: S3 Fig — Kidney weight/body weight (KW/BW) ratio from wild-type (circles) and heterozygous MITF-A transgenic (square symbol) mice (line 42) at birth (W0) and 3 (W3), 8 (W8), 16 (W16), and 24 (W24) weeks after birth. Because KW/BW data obtained from wild-type and transgenic mice showed the same profile in females and males, only data from females are shown. Data are means ± SEM (n = 3–7 per group). Mann Whitney test; transgenic versus wild-type: * P < 0.05, ** P < 0.01. (TIF) [file pgen.1007093.s006.tif]

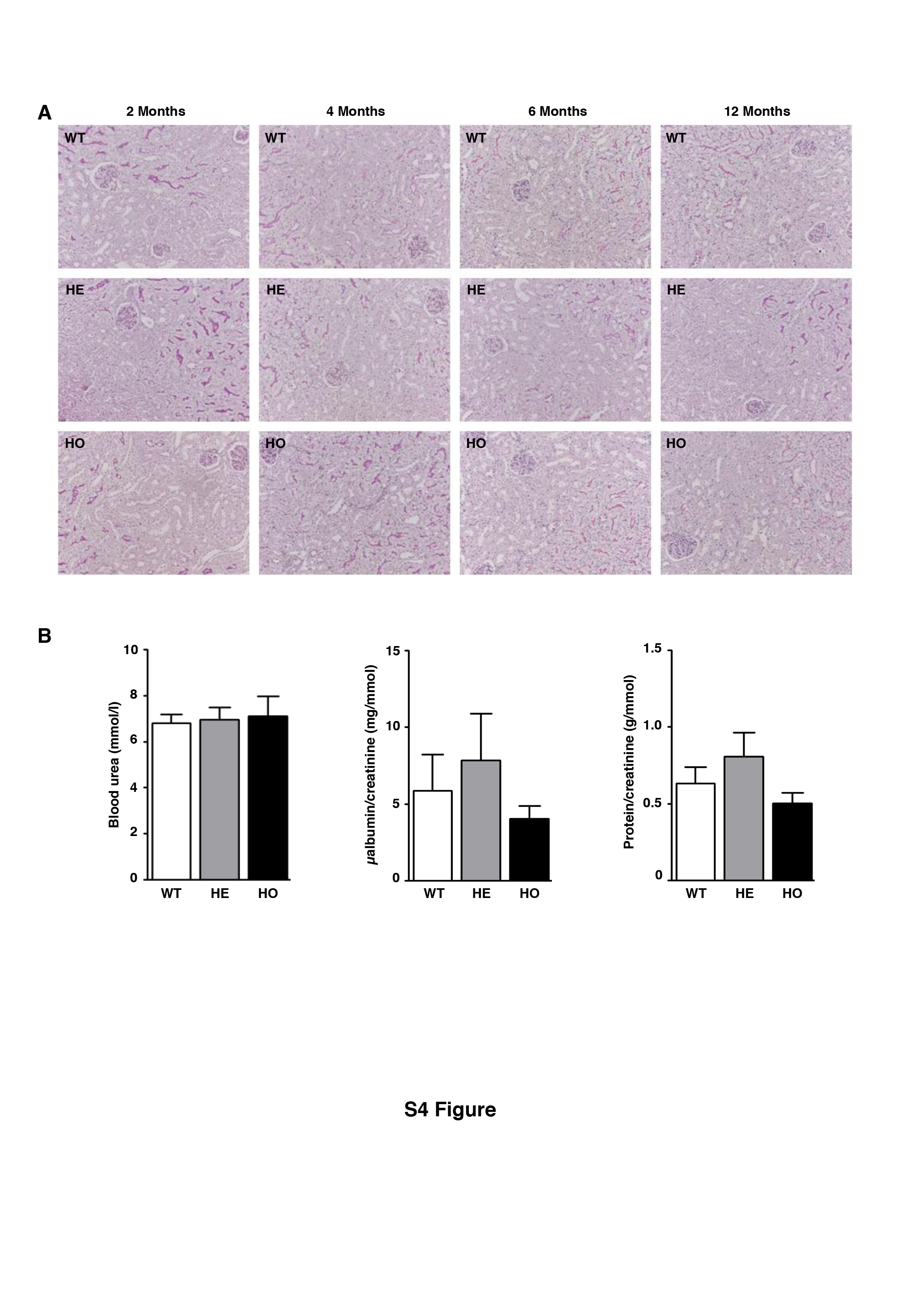

Supplement: S4 Fig — A) Kidney morphology from wild-type (WT), heterozygous (HE) and homozygous (HO) MITF-A transgenic mice (line 42) at 2, 4, 6 and 12 months after birth. PAS staining; magnification: X200. B) Plasma urea levels (left panel), urinary albumin/creatinine ratio (middle panel) and urinary protein/creatinine ratio (right panel) in WT, HE and HO MITF-A transgenic mice at 12 months. Because morphological and biological data from transgenic mice and wild type mice were indistinguishable between females and males, only data of female mice are shown. Any statistically significant difference was observed among the three experimental groups. (TIF) [file pgen.1007093.s007.tif]

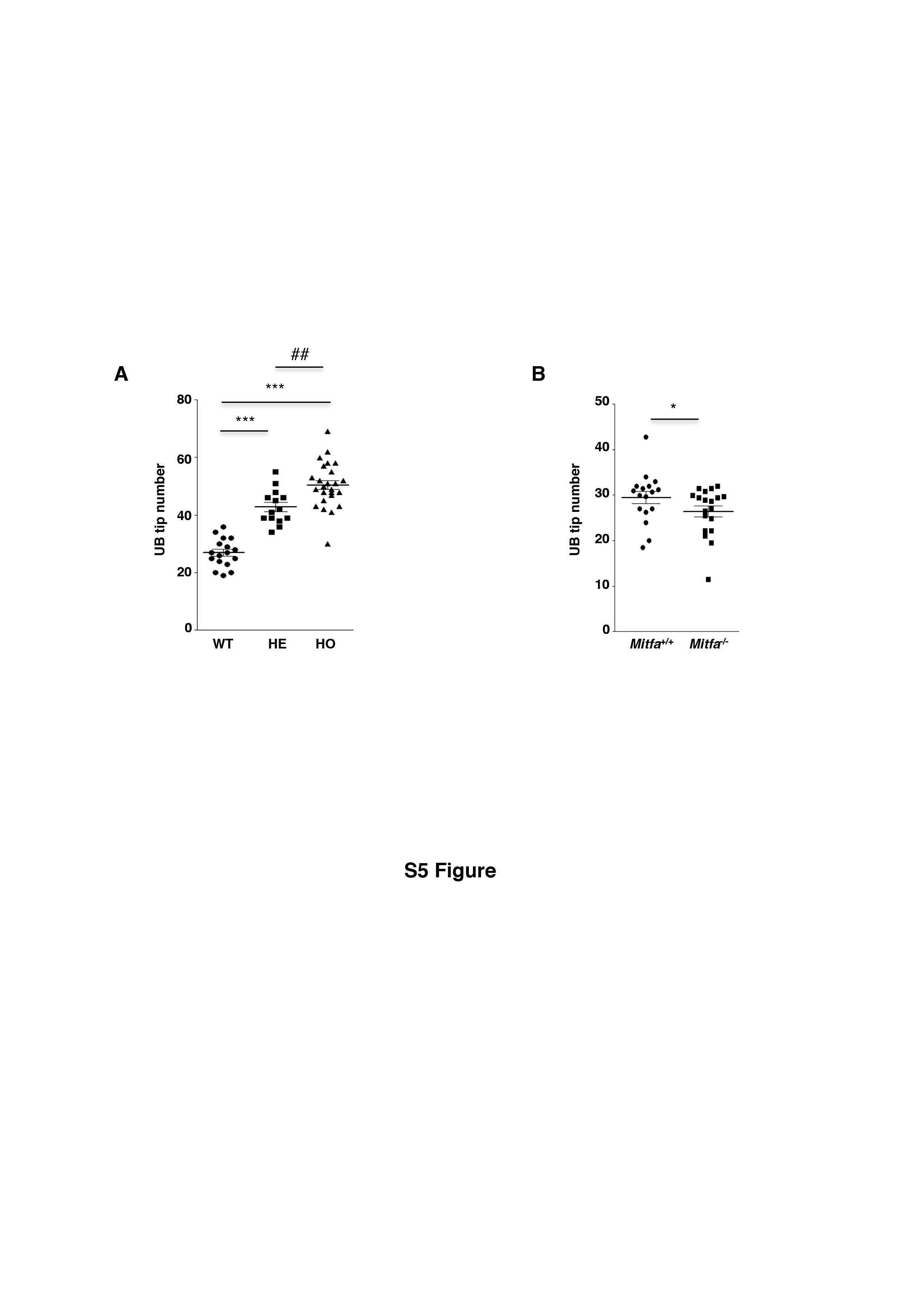

Supplement: S5 Fig — A) Ureteric bud (UB) branching, as assayed by counting the number of UB tips in wild-type (WT, n = 17), heterozygous (HE, n = 14) and homozygous (HO, n = 25) MITF-A transgenic metanephroi at E13.5. Data are means ± SEM. ANOVA followed by Tukey-Kramer test; transgenic versus wild-type mice: *** P < 0.001, HE versus HO MITF-A transgenic mice: ## P < 0.01. B) Number of UB tips in E13.5 kidneys from Mitfa+/+ (n = 15) and Mitfa-/- (n = 20) embryos. Data are means ± SEM. Mann-Whitney test; Mitfa-/- versus: Mitfa+/+: * P < 0.05. (TIF) [file pgen.1007093.s008.tif]

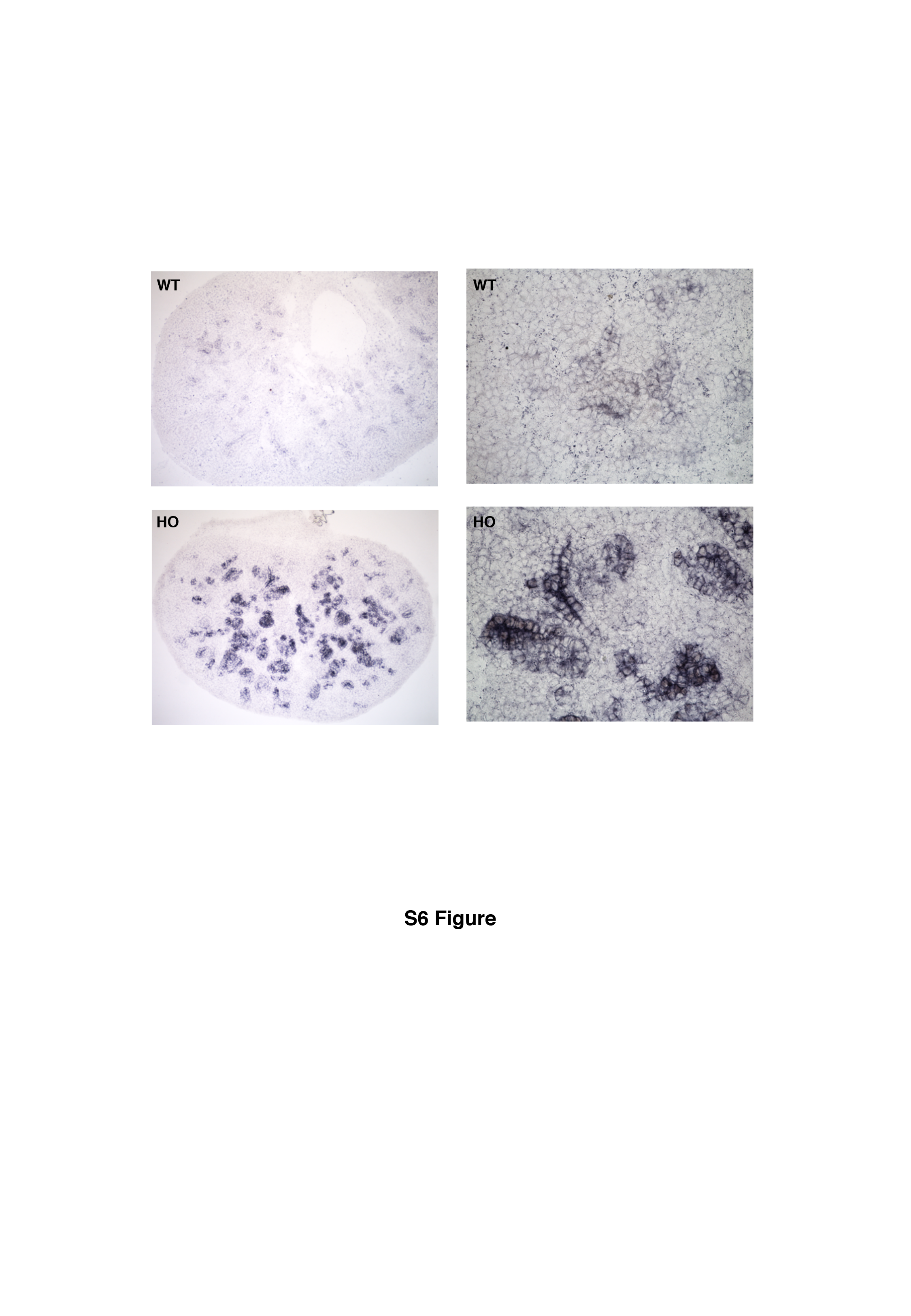

Supplement: S6 Fig — In situ hybridization of Mitf-A in kidneys of wild type (WT) and homozygous (HO) MITF-A transgenic embryos at E15.5. Note the strong staining in UB and early tubules in MITF-A transgenic kidneys. Magnifications: X100 (left panels) and X400 (right panels), n = 5–6 per genotype. (TIF) [file pgen.1007093.s009.tif]

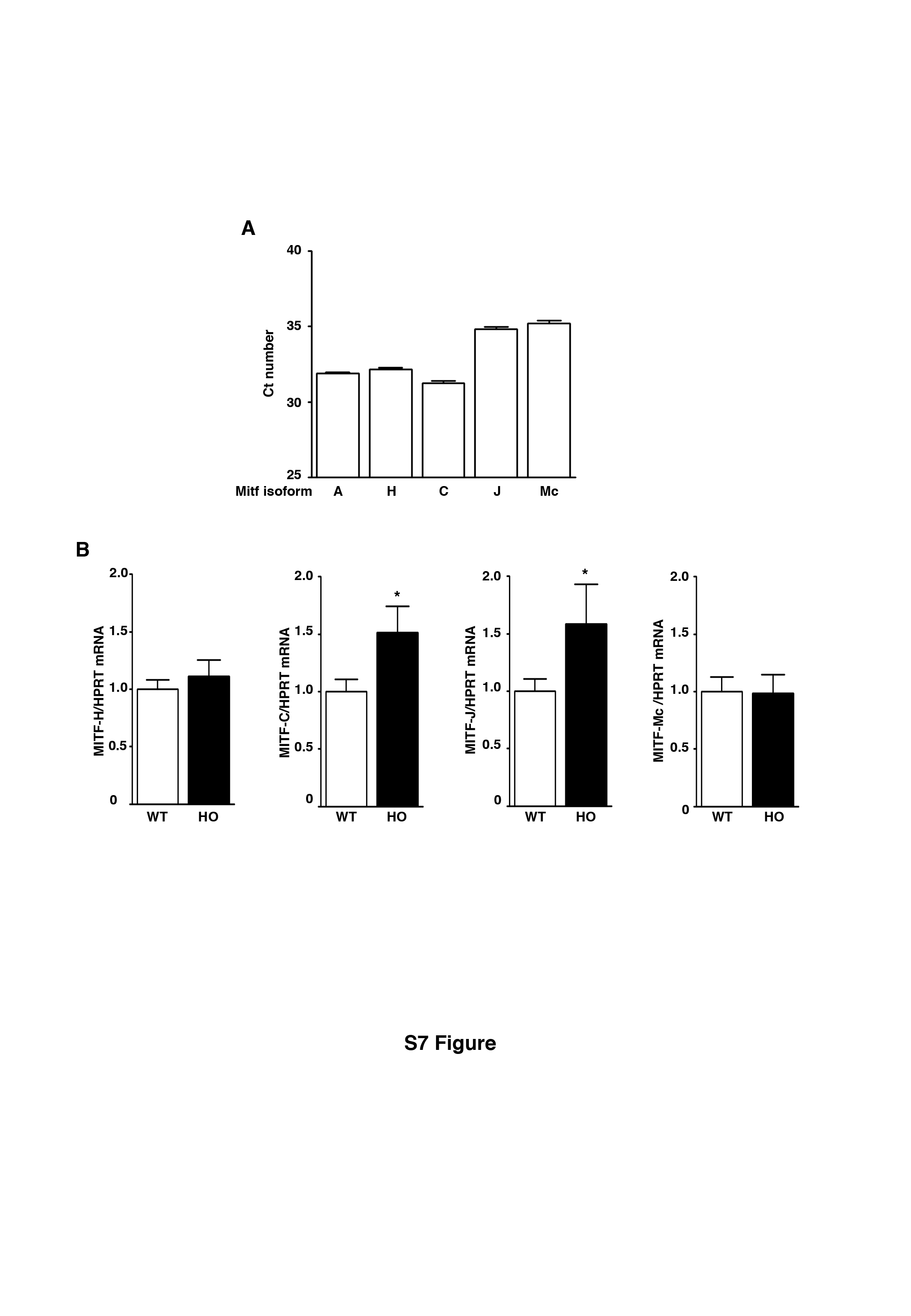

Supplement: S7 Fig — A) Mitf isoform expression levels in kidneys of wild-type embryos at E13.5 as judged by Ct values obtained from RT-PCR curves (Ct values inversely correlate with mRNA levels). Note that the other isoforms, namely Mitf-M, Mitf-B, Mitf-D and Mitf-E were undetectable in kidneys. B) Mitf-H, Mitf-C, Mitf-J and Mitf-Mc expression levels in kidneys of wild-type (WT) and homozygous (HO) transgenic embryos at E13.5. Data are means ± SEM; n = 5–6 per genotype. Mann Whitney test; transgenic versus wild-type mice: * P < 0.05. (TIF) [file pgen.1007093.s010.tif]

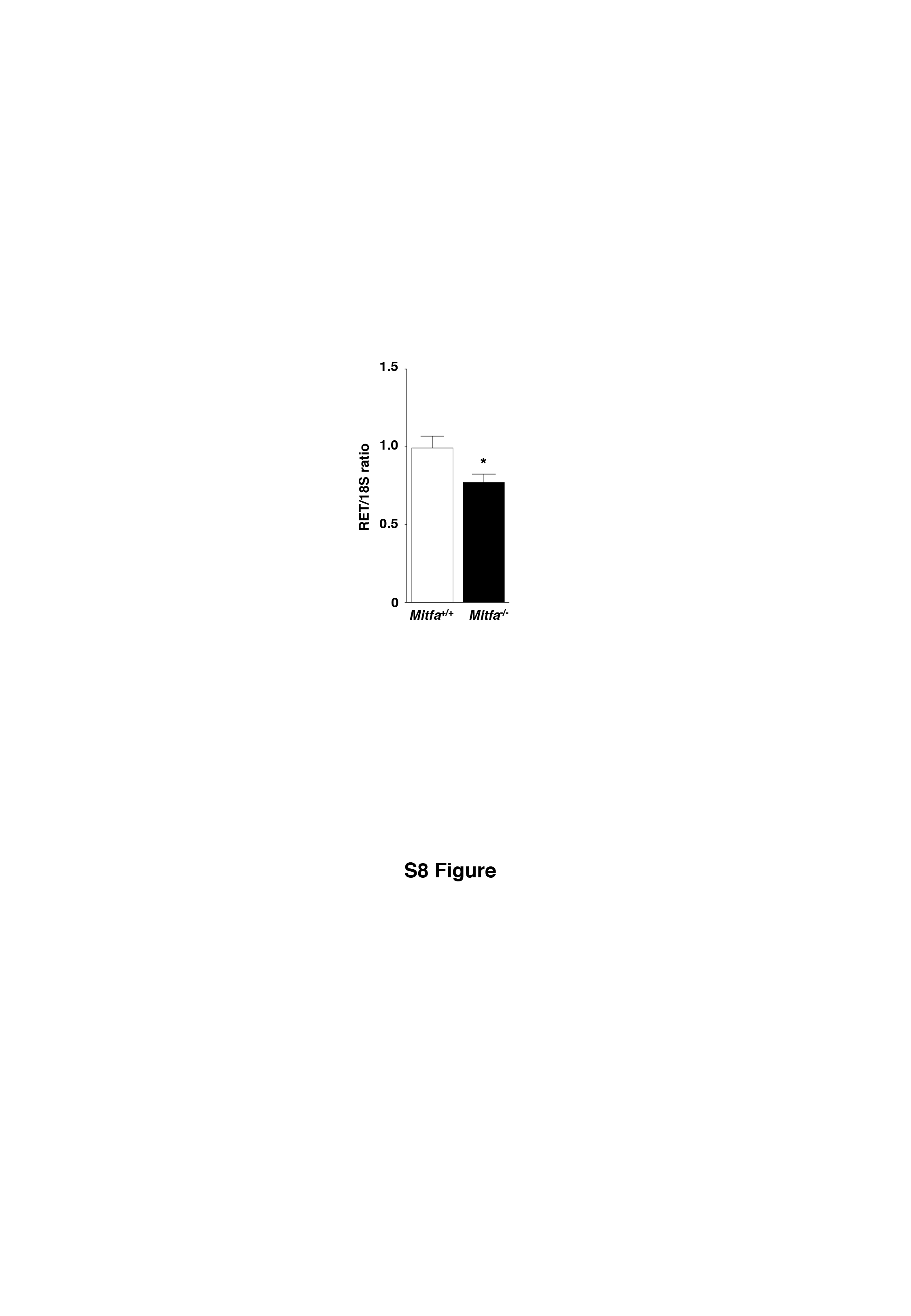

Supplement: S8 Fig — Ret mRNA expression evaluated by quantitative RT-PCR in kidneys from Mitfa+/+ and Mitfa-/- embryos at E13.5. Data are means ± SEM; n = 3–5 per genotype. Mann Whitney test; Mitfa+/+ versus Mitfa-/- mice: * P < 0.05. (TIF) [file pgen.1007093.s011.tif]

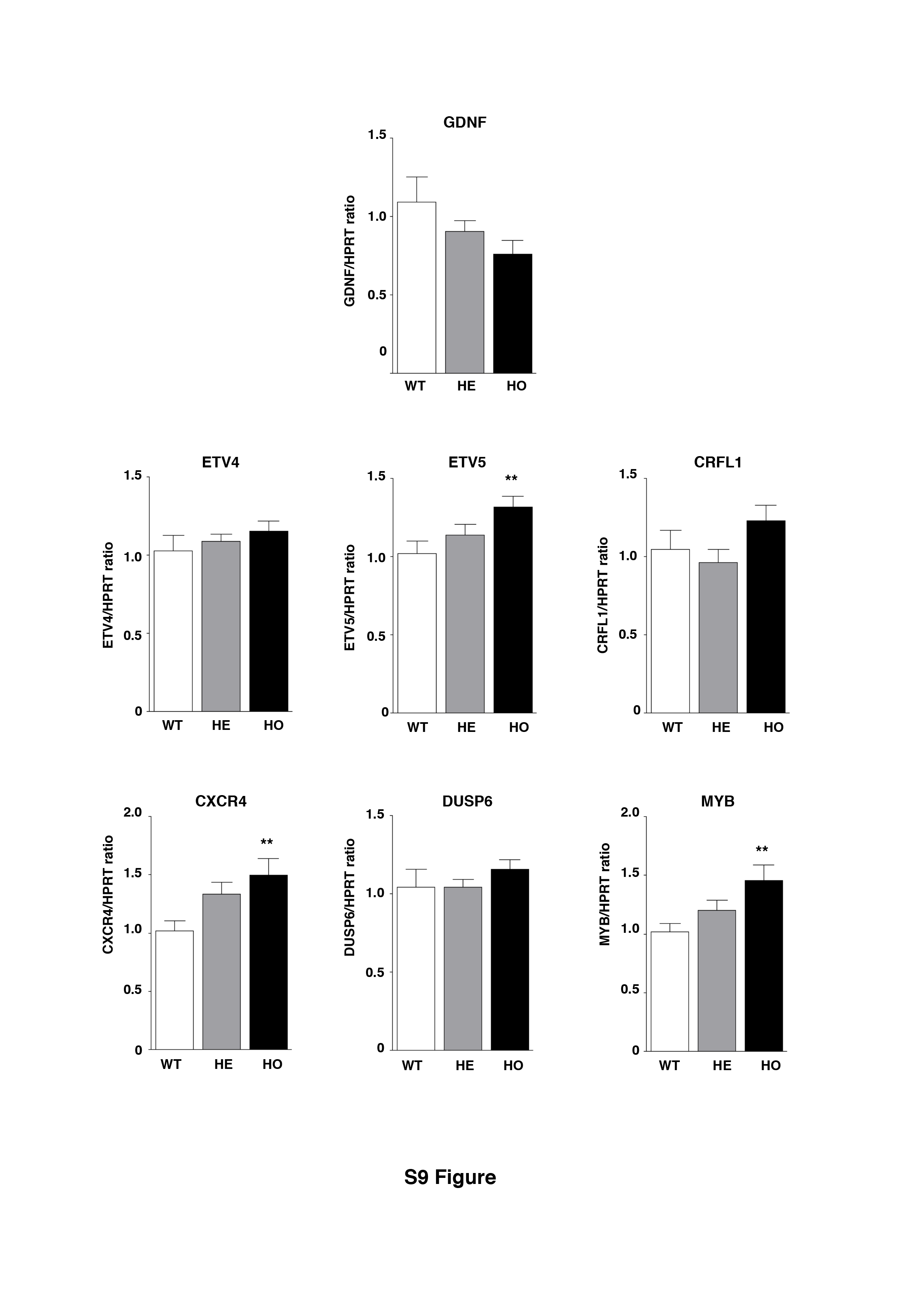

Supplement: S9 Fig — mRNA expression of known RET targets evaluated by quantitative RT-PCR in kidneys from wild-type (WT), heterozygous (HE) and homozygous (HO) MITF-A transgenic embryos at E13.5. Data are means ± SEM; n = 7–8 per genotype. ANOVA followed by Tukey-Kramer test; transgenic versus wild-type: ** P < 0.01. (TIF) [file pgen.1007093.s012.tif]
